# ﻿Description of three new bat-associated species of hard ticks (Acari, Ixodidae) from Japan

**DOI:** 10.3897/zookeys.1180.108418

**Published:** 2023-09-15

**Authors:** Ai Takano, Takeo Yamauchi, Mamoru Takahashi, Hiroshi Shimoda, Yasuhiro Gotoh, Junko Mizuno, Michio Natsume, Jenő Kontschán, Dávid Kováts, Vuong Tan Tu, Sándor Hornok

**Affiliations:** 1 Department of Veterinary Medicine, Joint Faculty of Veterinary Medicine and The United Graduate School of Veterinary Science, Yamaguchi University, Yamaguchi, Japan Yamaguchi University Yamaguchi Japan; 2 Laboratory of Entomology, Obihiro University of Agriculture and Veterinary Medicine, Obihiro, Hokkaido, Japan Obihiro University of Agriculture and Veterinary Medicine Obihiro Japan; 3 Department of Anesthesiology, Saitama Medical University, Saitama, Japan Saitama Medical University Saitama Japan; 4 Department of Bacteriology, Faculty of Medical Sciences, Kyushu University, Fukuoka, Japan Kyushu University Fukuoka Japan; 5 Natural Environmental Research Group, Gunma Prefecture, Gunma, Japan Natural Environmental Research Group Gunma Japan; 6 Department of Plant Sciences, Albert Kázmér Faculty of Mosonmagyaróvár, Széchenyi István University, Mosonmagyaróvár, Hungary Széchenyi István University Mosonmagyaróvár Hungary; 7 Hungarian Biodiversity Research Society, Budapest, Hungary Hungarian Biodiversity Research Society Budapest Hungary; 8 Institute of Ecology and Biological Resources, Vietnam Academy of Science and Technology, Hanoi, Vietnam Institute of Ecology and Biological Resources, Vietnam Academy of Science and Technology Hanoi Vietnam; 9 Graduate University of Science and Technology, Vietnam Academy of Science and Technology, Hanoi, Vietnam Graduate University of Science and Technology Hanoi Vietnam; 10 Department of Parasitology and Zoology, University of Veterinary Medicine, Budapest, Hungary University of Veterinary Medicine Budapest Hungary; 11 HUN-REN–UVMB Climate Change: New Blood-sucking Parasites and Vector-borne Pathogens Research Group, Budapest, Hungary HUN-REN–UVMB Climate Change: New Blood-sucking Parasites and Vector-borne Pathogens Research Group Budapest Hungary

**Keywords:** Chiroptera, *Eschatocephalus*, Ixodida, long-legged bat tick, mitochondrial, 16S rRNA gene

## Abstract

In Eurasia, the geographically most widespread ixodid tick species of the bat families Rhinolophidae Gray, Vespertilionidae Gray, and Miniopteridae Dobson were considered to belong to four species, *Ixodesvespertilionis* Koch, *I.collaris* Hornok, *I.ariadnae* Hornok, and *I.simplex* Neumann. Previous data attest that bat-associated tick species from Eastern Asia show remarkable genetic difference from the above four tick species, but in the absence of detailed morphological comparison these were regarded as conspecific. In this study we compensate for this lack of data on three bat-associated tick species, reporting their morphological comparison, as well as molecular and phylogenetic relationships. According to the results we describe the females of three tick species new to science, i.e., *I.nipponrhinolophi* Hornok & Takano, **sp. nov.**, *I.fuliginosus* Hornok & Takano, **sp. nov.**, and *I.fujitai* Hornok & Takano, **sp. nov.** In case of all three new tick species the cytochrome *c* oxidase subunit (coxI) gene showed remarkably high sequence differences from the species that they previously were thought to belong to, well exceeding the average limit delineating ixodid tick species. This, as well as observed morphological differences fully justify their taxonomical status as new species.

## ﻿Introduction

Bats (order Chiroptera) form the second largest order of mammals, with more than 1400 described species (Simmons and Cirranello 2023). Among mammals only bats are able to fly actively, which is an important factor behind their wide geographical distribution and seasonal migration (Hutterer et al. 2005). In part owing to their flight and their aggregated occurrence in colonies, bats frequently reach high population densities in or near urban habitats (Beltz 2018). During the past decades increasing chances of contact between bats and humans has been observed, due to increasing human introgression into natural bat habitats, as well as due to enhanced attraction of bats to agricultural facilities and human settlements where their food tends to abound (Han et al. 2015). Recent changes in bat behaviour also increase the risk of zoonotic transmission of pathogens by urbanisation as bats roost in artificial structures such as bridges and old mines, as well as homes, churches, schools, and barns (Beltz 2018). In addition, the total number of infectious bats may be higher in the urban, as opposed to rural, clusters (Plowright et al. 2011; Beltz 2018).

Bats play an important role in the epidemiology of vector-borne diseases. First and most importantly, the majority (approximately 70%) of all bat species are insectivorous (Simmons 2005) and due to this habit they have constant access to blood-sucking arthropod vectors as food items, possibly allowing mechanical transmission of relevant pathogens (Hornok et al. 2015a). Second, bats can also act as hosts of a broad range of haematophagous ectoparasites, among them biological vectors of pathogens (Klimpel and Mehlhorn 2014). Once infected with vector-borne pathogens, immunological adaptions of bats make them especially suitable reservoirs (Brook and Dobson 2015).

Among blood-sucking arthropod vectors, ticks (Acari: Ixodidae, Argasidae) are considered as the epidemiologically and ecologically most important in the temperate zone (Jongejan and Uilenberg 2004). Tick-infestation of bats may account for their infection with tick-borne pathogens, some of them zoonotic, as exemplified by borreliae, rickettsiae, and viruses (Oba et al. 2016; Beltz 2018). Bat ticks were also reported to carry DNA of pathogens (e.g., piroplasms) with high veterinary-medical importance (Hornok et al. 2016). In addition, the presence of ticks may affect the immunity to co‐infection of bats with other pathogens (Beltz 2018). Last but not least, among bat-specialist tick species, the soft tick *Cariosvespertilionis* Latreille (Argasidae), as well as two hard tick (Ixodidae) species, i.e., *Ixodesvespertilionis* Koch and *I.simplex* Neumann were also reported to opportunistically feed on humans (Jaenson et al. 1994; Piksa et al. 2013; Péter et al. 2021).

The taxonomy of bat-associated tick species has been subject to considerable revision during the past decade. First, a new ixodid bat tick species, *Ixodesariadnae* Hornok was discovered in Europe (Hornok et al. 2014), followed by the description of another new bat-specialist tick species, *Ixodescollaris* Hornok, 2016 from Asia. Although it was demonstrated that further “short and long-legged” ixodid bat tick species differ genetically enough to justify their taxonomic status as separate species (Hornok et al. 2015b), until now these were not described due to lack of sufficient candidates as type specimens, especially adults. Therefore, the most important driver of the present study was to examine females of bat-associated tick collected in Japan, in order to evaluate their morphology and (if substantiated) to describe them as new species.

## ﻿Materials and methods

### ﻿Sample collection and ethical permission

**Figure 1.**
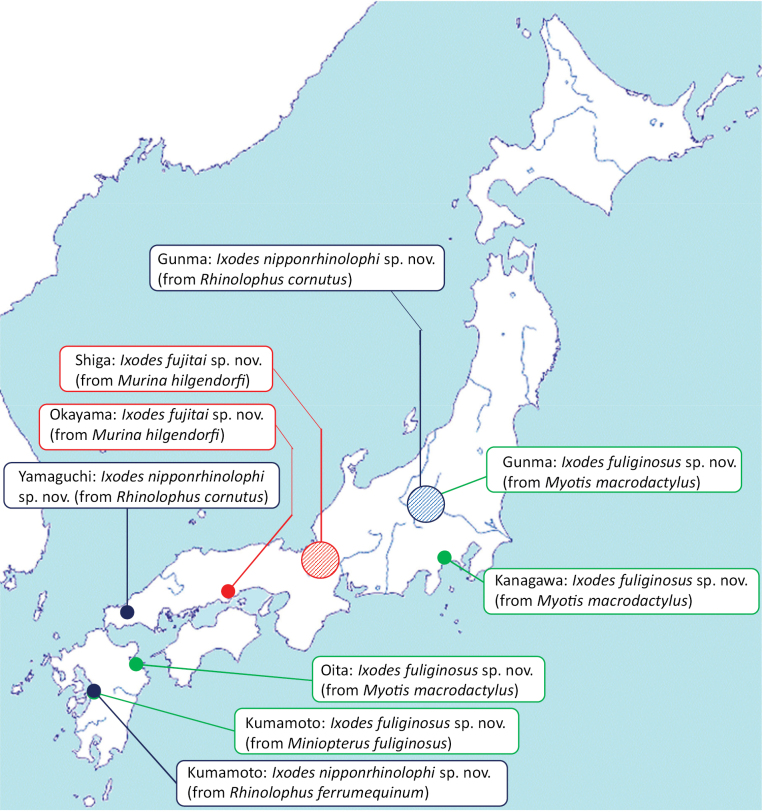
Map of Japan showing the origin of type specimens examined in this study. The blue spots indicate *Ixodesnipponrhinolophi* Hornok & Takano, sp. nov., the green-coloured spots mark locations of *Ixodesfuliginosus* Hornok & Takano, sp. nov., and red shows *Ixodesfujitai* Hornok & Takano, sp. nov. Larger shaded spots indicate the region of origin if the precise location was not known.

Ticks were removed from bats or cave walls at various locations in Japan (Fig. 1; type material is described below). Ethical permission for bat capture was provided in Yamaguchi Prefecture (Prefectural Government Approval No. R3-429-1 and R4-165-1). After collection, the ticks were stored individually in vials containing 70% or 96% ethanol.

### ﻿Morphological analyses

Previously or recently collected female ticks (Suppl. material 1), which were morphologically intact, were selected for description of new species. Measurements were performed and pictures were taken with a VHX-5000 digital microscope including the software VHX-H4M 3D (Keyence Co., Osaka, Japan). The sizes in the descriptions below are provided in millimetres.

In addition, for each new species, at least one recently collected adult tick was also used for DNA extraction from one leg or cutting the idiosoma ventrally (i.e., to preserve them as voucher specimens). By contrast, males, nymphs, and larvae available during the study period were not suitable for morphological description (i.e., their DNA was already extracted or were severely damaged). Therefore, these were only used for DNA extraction (from their whole body) and molecular phylogenetic analyses. Conspecificity of males, nymphs and larvae with morphologically analysed female(s) was confirmed by at least one mitochondrial marker. Type specimens (female ticks) are summarised in Table 1, whereas all ticks used for either morphological and/or molecular analyses are shown in Suppl. material 1.

**Table 1.** Female tick specimens used for descriptions and comparisons. For new species, the holotypes are in cells with light purple background; paratypes are in cells with light grey background.

Complex	Tick species	Source of origin	Sample code	Collected by	Country (region or location) of collection	Date collected: year-month-day
***I.vespertilionis***	*Ixodesnipponrhinolophi*	*Rhinolophuscornutus* ^4^	YB47	A.Takano	Japan (Mine city, Yamaguchi)	2021.02.25.
	*Ixodesnipponrhinolophi* ^1^	*Rhinolophusferrumequinum*	–	K. Funakoshi	Japan (Kuma-gun, Kumamoto)	1973.12.24.
	*Ixodesnipponrhinolophi* ^1^	*Rhinolophusferrumequinum*	–	K. Funakoshi	Japan (Kuma-gun, Kumamoto)	1979.04.12.
	*Ixodesnipponrhinolophi* ^1^	*Rhinolophusferrumequinum*	–	K. Funakoshi	Japan (Kuma-gun, Kumamoto)	1979.05.10.
	*Ixodesnipponrhinolophi* ^1^	*Rhinolophusferrumequinum*	–	K. Funakoshi	Japan (Kuma-gun, Kumamoto)	1979.05.25.
	*Ixodesnipponrhinolophi*	*Rhinolophuscornutus*	4673	M. Takahashi	Japan (Tano-gun, Gunma)	2005.04.23.
	*Ixodesvespertilionis*	Pálvölgyi Cave (wall)	KD36	D. Kováts	Hungary (Budapest)	2018.12.01.
	*Ixodesvespertilionis*	Leány Cave (wall)	CV1	D. Kováts	Hungary (Pilis Mountains)	2016.03.06.
	*Ixodesvespertilionis*	Leány Cave (wall)	CV142	D. Kováts	Hungary (Pilis Mountains)	2017.03.19.
	*Ixodescollaris* ^2^	*Hipposiderospomona*	VN14-0011	V. Tan Tu	Vietnam (Kon Tum)	2014.09.22.
***I.simplex***	*Ixodesfuliginosus*	*Myotismacrodactylus* ^5^	Kana2020	F. Sato	Japan (Sagamihara city, Kanagawa)	2022.09.30.
	*Ixodesfuliginosus* ^1^	*Miniopterusfuliginosus*	–	K. Funakoshi	Japan (Kuma-gun, Kumamoto)	1979.04.12.
	*Ixodesfuliginosus* ^1^	*Myotismacrodactylus*	–	K. Funakoshi	Japan (Hita-gun, Oita)	1997.07.17.
	*Ixodesfuliginosus*	*Myotismacrodactylus*	5997	M. Takahashi	Japan (Tone-gun, Gunma)	2014.06.17.
	*Ixodessimplex*	*Miniopterusschreibersii*	–	A. D. Sándor	Romania (Báziás)	2022.09.20-23.
***I.ariadnae***	*Ixodesfujitai*	unknown	–	H. Fujita	Japan (Inukami-gun, Shiga)	1990.12.09.
	*Ixodesfujitai* ^3^	*Murinahilgendorfi*	Iv_0ka2013	M. Yamada	Japan (Eniwa city, Okayama)	2013.03.09.
	*Ixodesfujitai*	*Murinahilgendorfi*	–	K. Okumura	Japan (Inukami-gun, Shiga)	2016.04.22.
	*Ixodesariadnae*	Legény Cave (wall)	CV86	D. Kováts	Hungary (Pilis Mountains)	2017.03.05.

^1^ Published in Yamauchi and Funakoshi 2000; ^2^ published in Hornok et al. 2016; ^3^ published in Hornok et al. 2015; ^4^ molted from a nymph collected from this host; ^5^ originally collected from this host, preserved after egg-laying.

### ﻿DNA extraction and coxI PCR analysis

DNA was extracted by DNeasy Blood and Tissue kit (QIAGEN, Germany). The partial fragments of cytochrome *c* oxidase subunit 1 gene (coxI), mitochondrial 16S rRNA gene (mt-rrs) and mitochondrial 12S rRNA gene (12S rDNA) were amplified using primers described in literature (Folmer et al. 1994; Ushijima et al. 2003; Beati and Keirans 2001; Norris et al. 1999). PCR was performed using Tks Gflex DNA Polymerase (TaKaRa Bio Inc., Japan). PCR products were purified with Axygen AxyPrep MAG PCR Clean-Up kit (Corning, USA) and sequenced by Eurofins Genomics Inc. (Tokyo, Japan). Obtained sequences were compared with GenBank data by the nucleotide BLASTN program (https://blast.ncbi.nlm.nih.gov).

### ﻿Mitochondrial genome sequencing

Mitochondrial genomes (mt-genome) were sequenced as previously described (Kelava et al. 2021). Briefly, long-range PCR was performed with mtG_K23 and mtG_K26 primer pair using PrimeSTAR GXL DNA Polymerase (TaKaRa Bio Inc.). Short PCRs for gap filling were performed using primer pair: mtG_GapF1 (5’-AGG AAG CTT AAA TTC CTC GCA T-3’) and mtG_GapR1 (5’-TGC CAG CCG CCG CGG TTA TAC A-3’). All PCR products were purified with Axygen AxyPrep MAG PCR Clean-Up kit (Corning) and sequenced by Illumina Miseq (Illumina, USA) for long-range PCR, and Eurofins Genomics Inc. for short-PCR products. The Illumina sequence libraries were constructed by NEBNext Ultra II FS DNA Library Prep Kit for Illumina (New England Biolabs) and were sequenced with the Illumina MiSeq platform with the paired-end sequence reads (300 bp ×2). The assembly was done by Platanus B ver. 1.3.2 (Kajitani et al. 2020). Mt-genome was annotated using Geneious Prime (Biomatters, Ltd., New Zealand). New sequences (LC769933–LC769956) were submitted to GenBank (Suppl. material 1).

### ﻿Phylogenetic analysis

The evolutionary history was inferred by using the Maximum Likelihood method, Kimura 2-parameter model with the MEGA version 7.0 software (Kumar et al. 2016). Sequence datasets were resampled 1,000 times to generate bootstrap values. The percentage of trees in which the associated taxa clustered together is shown next to the branches. Branch lengths are measured in the number of substitutions per site. The analysis involved 40 nucleotide sequences, and there were a total of 578 positions in the final dataset.

### ﻿Depositories

The holotypes were used for measurements and (together with paratypes) for illustrations. The holotypes will be deposited in the Center for Collections, National Museum of Nature and Science, Tokyo, Japan. The paratypes will remain in the collections of Takeo Yamauchi and Mamoru Takahashi (Table 1).

## ﻿Taxonomic account

### ﻿Family Ixodidae Koch

**Genus *Ixodes* Latreille**

**Subgenus Eschatocephalus von Frauenfeld**

#### ﻿ Ixodes nipponrhinolophi

Taxon classificationAnimaliaIxodidaIxodidae

Hornok & Takano sp. nov.

DFE903F8-00CD-5F20-A049-33E71BAB2C4A

https://zoobank.org/08621B6E-5F55-44DB-8DBE-C3B9C5363558

Figs 2 , 3

##### Diagnosis.

Medium size (female 4 mm long) brown tick. Legs and palps long. Scutum posteriorly broad, rounded, with moderately deep cervical grooves and pits. Hair covering dense both dorsally and ventrally. Coxae without spurs, coxae I and II with straight and III and IV with semicircular medial edges. Spiracular plates subcircular.

##### Material examined.

***Holotype***: female, from *Rhinolophuscornutus* Temminck, Japan, Yamaguchi, Mine city, 34.251084°N, 131.243056°E (DD), 25 February 2021, A. Takano coll. ***Paratypes***: five females. (1): from *Rhinolophusferrumequinum* (Schreber), Japan, Kumamoto, Kuma-gun, 32.252183°N, 130.651239°E (DD), 24 December 1973, K. Funakoshi coll. (2): female, from *Rhinolophusferrumequinum*, Japan, Kumamoto, Kuma-gun, 32.252183°N, 130.651239°E (DD), 12 April 1979, K. Funakoshi coll. (3): female, from *Rhinolophusferrumequinum*, Japan, Kumamoto, Kuma-gun, 32.252183°N, 130.651239°E (DD), 10 May 1979, K. Funakoshi coll. (4): female, from *Rhinolophusferrumequinum*, Japan, Kumamoto, Kuma-gun, 32.252183°N, 130.651239°E (DD), 25 May 1979, K. Funakoshi coll. (5): female, from *Rhinolophuscornutus*, Japan, Gunma, Tano-gun, 36.086915°N, 138.721945°E (DD), 23 April 2005, M. Takahashi coll.

##### Morphology of female (holotype, unengorged).

Length of the idiosoma (from the half point between scapular apices to the middle of posterior margin) 3.18, width 1.74, ratio of idiosomal length/width 1.83 (Suppl. material 2: fig. 1A).

**Figure 2.**
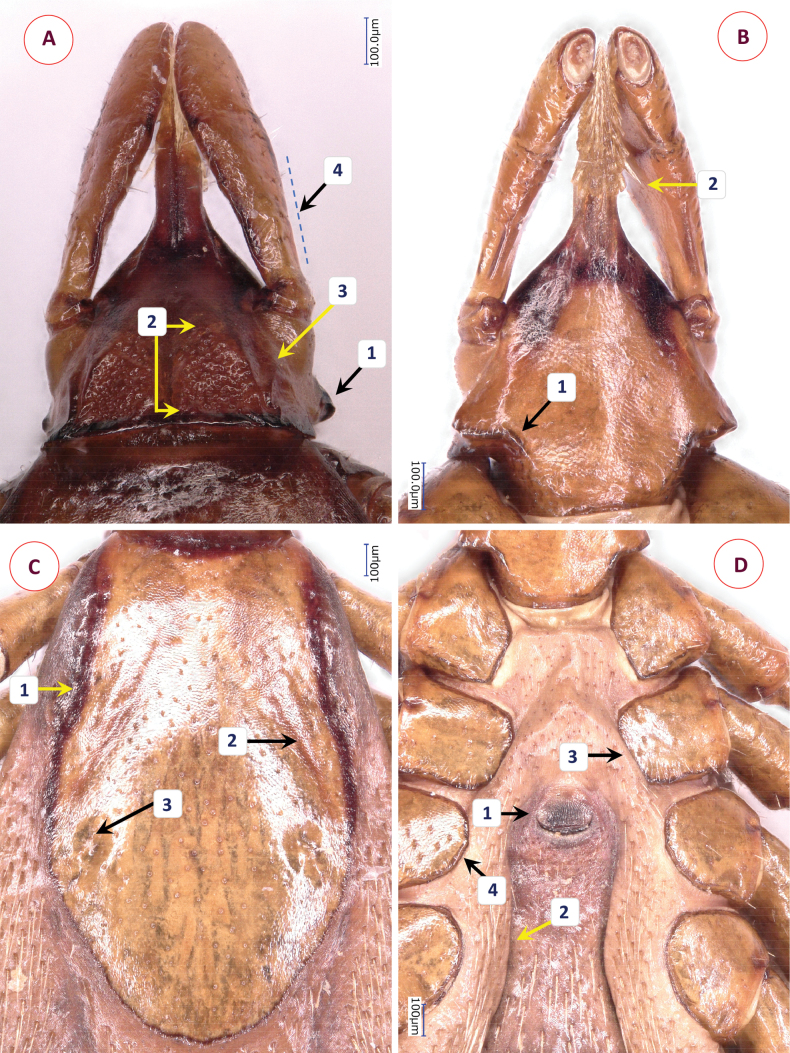
The morphology of *Ixodesnipponrhinolophi* Hornok & Takano, sp. nov.: **A** dorsal aspect of basis capituli **B** ventral aspect of basis capituli **C** scutum **D** idiosoma ventrally. Numbers are referred to in the text where relevant structures are described. Scale bars: 100 μm.

**Figure 3.**
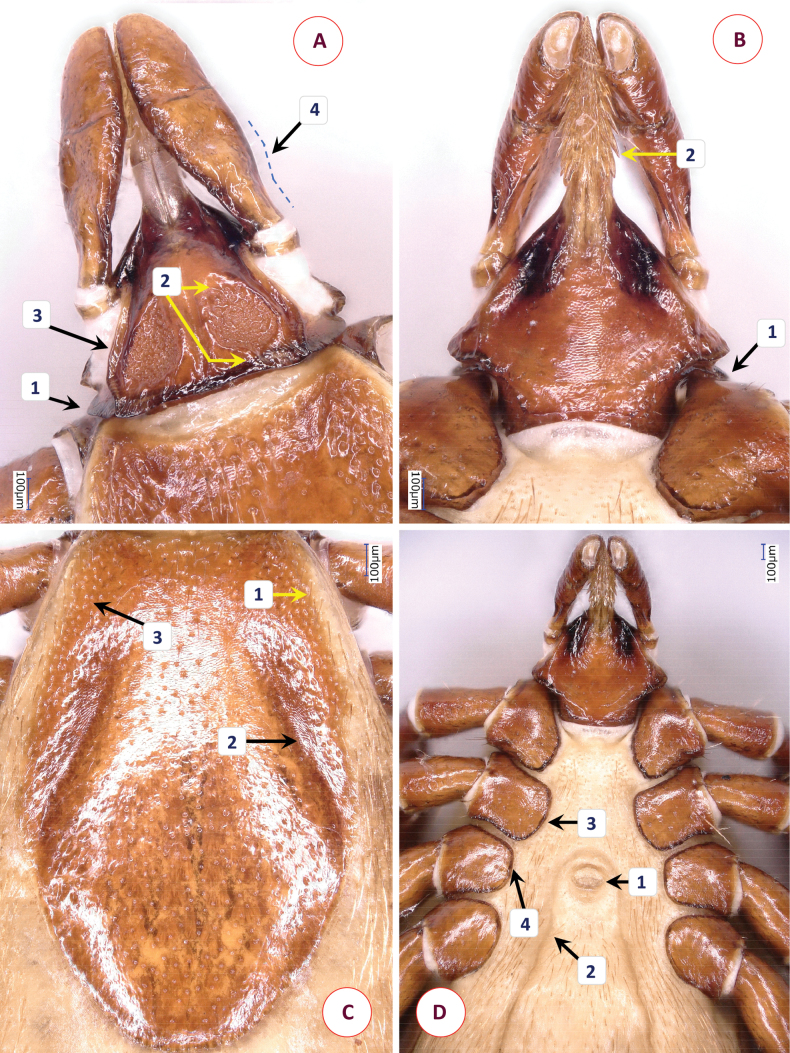
The morphology of *Ixodescollaris*: **A** dorsal aspect of basis capituli **B** ventral aspect of basis capituli **C** scutum **D** idiosoma ventrally. Numbers are referred to in the text where relevant structures are described. Scale bars: 100 μm.

Scutum elongated, tie-shaped, broadest near posterior third. Deepest point of concavity at anterior third of length (Fig. 2C–1). Length of scutum 1.62, maximum width 0.99, ratio of length/width 1.64. Scutum with cervical grooves deep, narrow in second quarter of length (Fig. 2C–2); scattered, small punctuations; and two pits close to its maximum width (Fig. 2C–3). Posterolateral edge (after maximum width) convex. Scutal setae few, close to scapulae 0.03 long.

Alloscutum with dense hair covering dorsally. Length of centrodorsal setae 0.1, marginodorsal setae 0.16. Idiosoma has dense hair covering ventrally. Genital aperture broad U-shaped, with nearly parallel end (Fig. 2D–1), slightly posterior to 2^nd^-to-3^rd^ intercoxal space. Genital groove posteriorly converging, then diverging (Fig. 2D–2), bowling pin-shaped (Suppl. material 2: fig. 1B). Spiracular plates subcircular, diameter 0.44, aeropyles randomly distributed, width of aeropyle rows (minimum to maximum) 2:7; position of spiracle opening submarginal, rounded, diameter 0.06 (Suppl. material 2: fig. 1C). Anal valves with 4-4 setae, arranged in C-shaped curve (Suppl. material 2: fig. 1D). Anal groove slightly converging (Suppl. material 2: fig. 1B).

Length of gnathosoma (from palpal apices to posterior margin of basis capituli) 0.84, width of basis capituli dorsally 0.63. Ratio of gnathosomal length/basis capituli width 1.33. Basis capituli triangular, its sides anteriorly converging, broadest at lateral ridge continuing ventrally (Fig. 2A–1), posteriorly as broad as at maximum width of palpal base, without cornuae; posterior margin dark, sclerotised, nearly linear. Shape of areae porosae triangular anteriorly, broad posteriorly (Fig. 2A–2), with short, low lateral ridge (Fig. 2A–3). Ventrally the basis capituli triangular anterior to maximum width, reverse trapezoidal posteriorly, with squared concavity in between. Auriculae absent (Fig. 2B–1).

**Figure 4.**
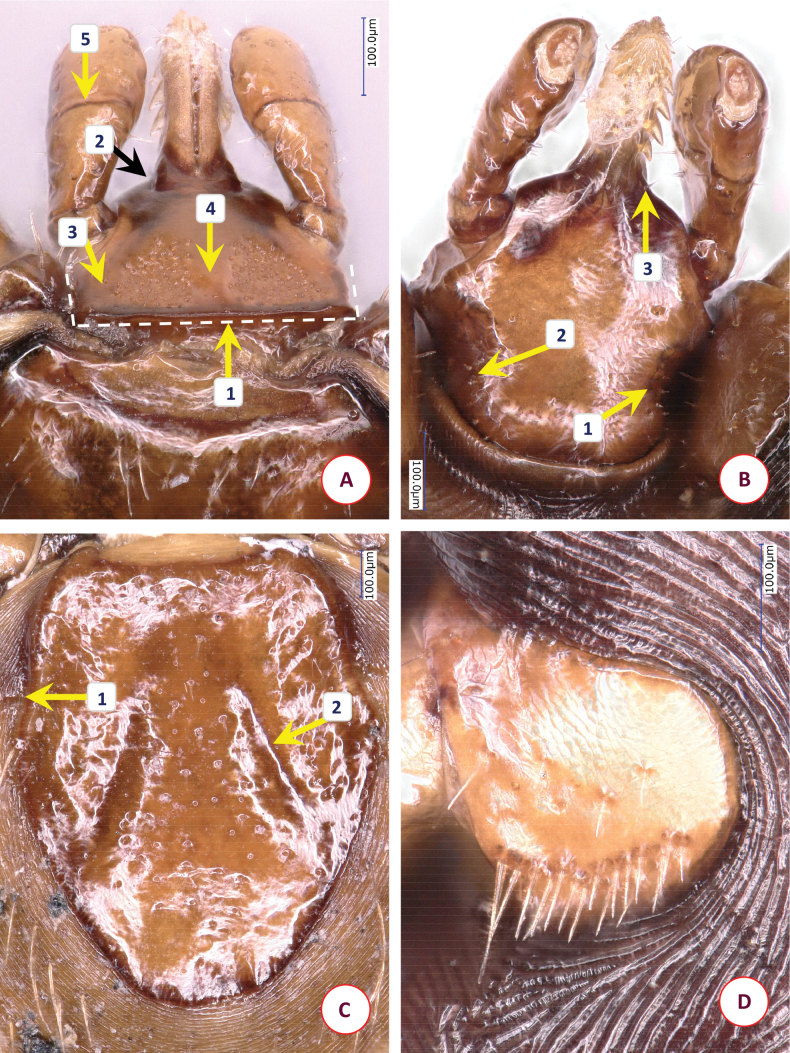
The morphology of *Ixodesfuliginosus* Hornok & Takano, sp. nov.: **A** dorsal aspect of basis capituli **B** ventral aspect of basis capituli **C** scutum **D** coxa IV. Numbers are referred to in the text where relevant structures are described. Dashed line indicates the edge of basis capituli. Scale bars: 100 μm.

Palps (dorsal view) long, club-shaped, length 0.7, broadest anterior to junction of segments II and III, maximum width 0.17, ratio of length/width 4.1. Palpal hairs 0.03–0.08, longest medially on palpal segment II. Palpal segment I. 0.08, palpal segment II. 0.4, palpal segment III. 0.23 long. Ratio of palpal segments II/III 1.8, segment II 2.8× longer than broad when viewed vertically; its “stalk” with surface in level with that of broad part (Fig. 2A–4).

Hypostome lanceolate, anterior tapering straight, apex pointed. Length 0.4, width 0.18, ratio of length/width 2.2. Dental formula 3/3 (mid-length), apically 4/4 or more. Teeth posteriorly long (0.06), slender, elevated (Fig. 2B–2).

Legs long and slender. All coxae marginally dark (sclerotised), without spines or spurs. Coxa I trapezoid, with minute hairs (posteriorly 0.05). Coxa II rectangular, medial edge straight (Fig. 2D–3), posteriorly wrinkled, posterolateral hairs short (0.1). Coxa III semicircular (Fig. 2D–4), hairs 0.03–0.07. Coxa IV semicircular, hairs 0.05. Tarsus I with long setae (< 0.25) dorsally, length 1.3, maximum diameter 0.15, ratio of length/diameter 8.7. Haller’s organ elongated, with grouped (non-linear) anterior pit sensillae, one of them longer (0.04).

##### Differential diagnosis.

*Ixodesnipponrhinolophi* Hornok & Takano, sp. nov. is easily distinguished from members of the *Ixodessimplex* complex based on the long legs, and from members of the *Ixodesariadnae* complex according to its long palps. Within the *Ixodesvespertilionis* complex, differences in comparison with females of the most similar species, *Ixodescollaris* include the following characters of the latter. Anteriolaterally on the scutum, the deepest point of concavity is at the anterior 1/8 of its length (Fig. 3C–1); cervical grooves broad (Fig. 3C–2), there are no pits; punctuations dense, especially anteriolaterally, (Fig. 3C–3). Genital aperture horizontally C-shaped, with diverging end (Fig. 3D–1), between third coxae, genital groove posteriorly diverging (Fig. 2D–2; Suppl. material 3: fig. 2B). Spiracle opening elongated, diameter 0.1 (Suppl. material 4: fig. 3C). Anal groove parallel behind the anus (Suppl. material 3: fig. 2D). Basis capituli posteriorly with transverse ridge, continuing laterally then ventrally as broad “collar” (Fig. 3A–1); shape of areae porosae elongated, tapering both anteriorly and posteriorly (Fig. 3A–2), their border well-defined, elevated laterally as diverging, then converging high ridge (Fig. 3A–3). Ventrally collar broad, extending above first coxae parallel with their surface (Fig. 3B–1), unlike in *I.nipponrhinolophi* Hornok & Takano, sp. nov. Ratio of palpal segments II to III 1.6, segment II only 2.1-times longer than broad when viewed vertically; its broad part with surface elevated above that of “stalk” (Fig. 3A–4). Hypostome teeth posteriorly less elevated (Fig. 3B–2). Medial edge of coxa II slightly curved (Fig. 3D–3), of coxa III straight (Fig. 3D–4).

##### Gene sequences.

The complete mitochondrial genome sequence was deposited in GenBank (LC769935). All accession numbers relevant to the new species are listed in Suppl. material 1.

##### Host records.

Known host species: *Rhinolophuscornutus*, *Rhinolophusferrumequinum*.

##### Etymology.

The name of the new species refers to Japan (in Japanese: Nippon), where all specimens have been collected, and to the type host family of horseshoe bats, Rhinolophidae.

#### ﻿ Ixodes fuliginosus

Taxon classificationAnimaliaIxodidaIxodidae

Hornok & Takano sp. nov.

27946E21-6E79-5AF6-A84C-5BDB51640297

https://zoobank.org/70478C08-894B-44EF-B307-40982FA22EFD

Figs 4 , 5

##### Diagnosis.

Medium size (female 3.3 mm long) brown tick. Legs and palps short. Scutum oval, shield-shaped. Hair covering dense dorsally but sparse ventrally. Coxae without spurs. Coxa IV with 16–18 hairs posteriorly. Spiracular plates ovoid, somewhat triangular.

##### Material examined.

***Holotype***: female, from *Myotismacrodactylus* Temminck, Japan, Kanagawa, Sagamihara city, 35.623170°N, 139.165542°E (DD), 30 September 2022, F. Sato coll. ***Paratypes***: three females. (1): from *Miniopterusfuliginosus* Hodgson, Japan, Kumamoto, Kuma-gun, 32.252183°N, 130.651239°E (DD), 12 April 1979, K. Funakoshi coll. (2): female, from *Myotismacrodactylus*, Japan, Oita, Hita-gun, 33.228090°N, 130.981712°E (DD), 17 July 1997, K. Funakoshi coll. (3): female, from *Myotismacrodactylus*, Japan, Gunma, Tone-gun, 36.685602 °N, 138.925637°E (DD), 17 June 2014, M. Takahashi coll.

##### Morphology of female (holotype, engorged).

Length of the idiosoma (from the half point between scapular apices to the middle of posterior margin) 3.2, width 2.1, ratio of idiosomal length/width 1.5.

Scutum oval, shield-shaped, anteriolaterally concave (Fig. 4C–1; Suppl. material 4: fig. 3A), broadest slightly anteriorly to its mid-length. Mid-length of scutum 0.95, maximum width 0.79, ratio of length/width 1.2. Maximum width of scutum to interscapular distance ratio 1.86. Cervical grooves straight, broad, directed to mid-posterolateral edge (Fig. 4C–2). On the scutum scarce punctuations, except in cervical fields. Posterior edge rounded. Scutal setae medially, anteriolaterally 0.04–0.05.

Alloscutum has dense hair covering dorsally. Length of centrodorsal setae 0.13, marginodorsal setae 0.1 (near peritreme). Idiosoma has sparse hair covering ventrally. Genital aperture straight, between 2^nd^ coxae (Suppl. material 4: fig. 3B-1). Genital grooves narrowest (as a “waist”) between 3^rd^ and 4^th^ intercoxal spaces (Suppl. material 4: fig. 3B-2), slightly diverging (almost parallel) to mid-length between genital opening and anus, then sharply diverging. Spiracular plates ovoid, diameter 0.24, aeropyles in rows (minimum to maximum) of 3–8; position of spiracle opening submarginal, rounded, diameter 0.03 (Suppl. material 4: fig. 3C). The shape of spiracular plates may have individual variation (Suppl. material 4: fig. 3D). Anal groove curved at front, slightly diverging behind the anus (Suppl. material 4: fig. 3B-3).

Length of gnathosoma (from palpal apices to posterior margin of basis capituli) 0.47, width of basis capituli dorsally 0.35. Ratio of gnathosomal length to basis capituli width 1.34. Basis capituli pentagonal, its sides parallel, posterior margin nearly straight (Fig. 4A–1), without cornuae and posteriolateral corner blunt. Basis length 0.16, width 0.37, length to width ratio 0.43. Front of basis nearly horizontal when joining the hypostome, enclosing an angle of ~ 120° (Fig. 4A–2). Shape of areae porosae triangular, their surface in level with surrounding smooth surface of basis (Fig. 4A–3). Laterally to areae porosae broad, smooth margin, similar in width (0.05) to interval between them (Fig. 4A–4). Ventrally basis capituli broad both anteriorly and posteriorly, with a narrowing (“waist”) between (Fig. 4B–1), thus forming a figure-of-eight shape, posteriolaterally with hair covering (length 0.01) (Fig. 4B–2). Auriculae absent, auricular ridges short and inapparent. Posthypostomal setae medially, laterally 1-1 pair (length 0.02) (Fig. 4B–3).

**Figure 5.**
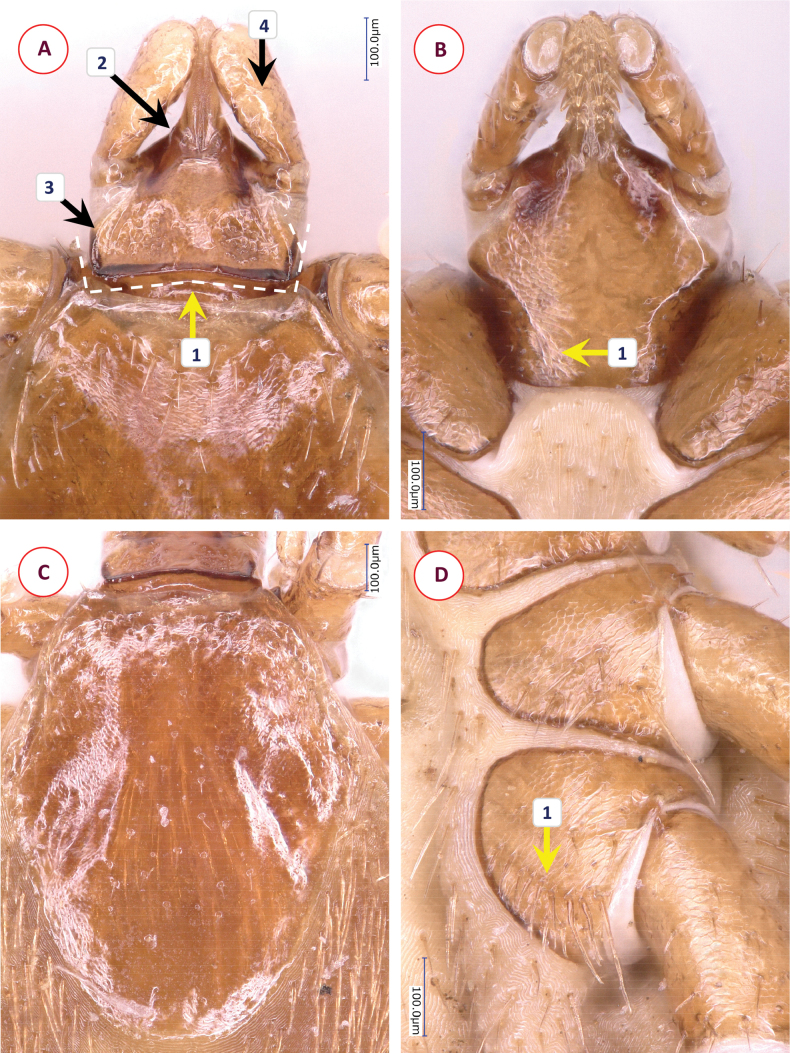
The morphology of *Ixodessimplex*: **A** dorsal aspect of basis capituli **B** ventral aspect of basis capituli **C** scutum **D** coxae III and IV. Numbers are referred to in the text where relevant structures are described. Dashed line indicates the edge of basis capituli. Scale bars: 100 μm.

Palps (dorsal view) short, club-shaped, anteriorly rounded, edge curved medially, straight laterally, length 0.33, maximum width 0.13, ratio of length/width 2.5. Joining of palpal segments II and III clearly visible as dark line (Fig. 4A–5). Palpal hairs few, numbering five or six laterally, dorsally, and medially; anteriorly short (0.015), longest medially on segment II (0.035). Palpal segment II triangular, 0.14 long. Palpal segment III same length (0.14).

Hypostome apex blunt, length 0.2, width 0.1, ratio of length/width 2. Dental formula 2/2 distally, 3/3 proximally (2-2 rows).

Legs short. All coxae medially rounded, without spines or spurs. Coxae I trapezoid, with three hairs (length 0.06–0.13). Coxa II with three hairs, coxa III with eight hairs, coxa IV with a tuft of 16–18 posterior hairs (0.035–0.125) (Fig. 4D). Tarsus I. length 0.73, maximum diameter 0.16, length to diameter ratio 4.5. Tarsal hairs short (0.03–0.05), except one long dorsal pair (0.17).

##### Differential diagnosis.

*Ixodesfuliginosus* Hornok & Takano, sp. nov. can be easily distinguished from members of the *I.vespertilionis* and *I.ariadnae* complexes based on its short legs (length/maximum diameter ratio below 5, vs above 8 in long-legged bat ticks). Differences in comparison with females of the most similar species, *Ixodessimplex* include the following characters of the latter. More elongated, rhombus-shaped scutum (Suppl. material 5: fig. 4A), with length/width ratio 1.3 or above (vs ~ 1.2 in *I.fuliginosus* Hornok & Takano, sp. nov.) (Fig. 5C). Maximum width of scutum/interscapular distance ratio 2.16 (vs 1.86 in *I.fuliginosus* Hornok & Takano, sp. nov.). Position of genital aperture more posterior, between 3^rd^ and 4^th^ intercoxal spaces (Suppl. material 5: fig. 4B-1), and genital groove narrowest between 4^th^ coxae (Suppl. material 5: fig. 4B-2). Long hair (0.1) anterior to and encircling the genital pore of *I.simplex* (Suppl. material 5: fig. 4B), absent on holotype and paratypes of *I.fuliginosus* Hornok & Takano, sp. nov. Basis capituli sides forwardly diverging then converging, posterior edge concave (Fig. 5A–1), without cornuae but posteriolateral corner sharp. Basis length 0.22, width 0.33, length to width ratio 0.67 (i.e., basis more elongated in *I.simplex* than in *I.fuliginosus* Hornok & Takano, sp. nov.). Front of basis steeply sloping when joining the hypostome, enclosing an angle larger (~ 135°) in *I.simplex* than in *I.fuliginosus* Hornok & Takano, sp. nov. (Fig. 5A–2). In *I.simplex* lateral margins of areae porosae elevated as ridge (Fig. 5A–3), much narrower than interval between areae, whereas in *I.fuliginosus* Hornok & Takano, sp. nov. lateral margins are smooth and broad. The separation of palpal segments II and III indistinct in *I.simplex* (Fig. 5A–4), unlike in *I.fuliginosus* Hornok & Takano, sp. nov. Ventrally the basis is rhombus-shaped, posteriorly converging in *I.simplex* (Fig. 5B–1) unlike in *I.fuliginosus* Hornok & Takano, sp. nov. The number of posterior setae (hairs) is fewer, 8–12 on coxae IV in *I.simplex* (Fig. 5D–1). The spiracular plates and the slightly diverging anal grooves are similar in these two species (Suppl. material 5: fig. 4C, D).

##### Gene sequences.

Complete mitochondrial genome sequence was deposited in the GenBank (LC769933). All accession numbers relevant to the new species are listed in Suppl. material 1.

##### Host records.

Known host species: *Miniopterusfuliginosus*, *Myotismacrodactylus*.

##### Etymology.

The name of the new species refers to its bat host species of the genus *Miniopterus* which occurs in Japan (the type species of the *I.simplex* complex to which *I.fuliginosus* Hornok & Takano, sp. nov. belongs is a specific parasite of this genus of bats).

#### ﻿ Ixodes fujitai

Taxon classificationAnimaliaIxodidaIxodidae

Hornok & Takano sp. nov.

564ED6C4-E7DC-5B3D-A0D3-E83A8C61B4BB

https://zoobank.org/AFE18817-243E-4793-B1E3-D49C02D7C7C8

Figs 6 , 7

##### Diagnosis.

Medium size (engorged female 6.8 mm long) yellowish tick. Legs long, palps short, areae porosae large. Scutum anteriorly trapezoid, posterolateral edge straight then rounded convex with wrinkled surface along its margin. Hair covering is sparse both dorsally and ventrally.

##### Material examined.

***Holotype***: female, from unknown host species, Japan, Shiga, Inukami-gun, 35.222448°N, 136.291747°E (DD), 9 December 1990, H. Fujita coll. ***Paratypes***: two females. (1): from *Murinahilgendorfi* Peters, Japan, Okayama, Eniwa city, 34.961817°N, 133.631483°E (DD), 3 September 2013, K. Funakoshi coll. (2): female, from *Murinahilgendorfi*, Japan, Shiga, Inukami-gun, 35.222448°N, 136.291747°E (DD), 22 April 2016, K. Okumura coll.

##### Morphology of female (holotype, engorged).

Length of the idiosoma (from the half point between scapular apices to the middle of posterior margin) 6.8, width 4.8, ratio of idiosomal length/width 1.42.

**Figure 6.**
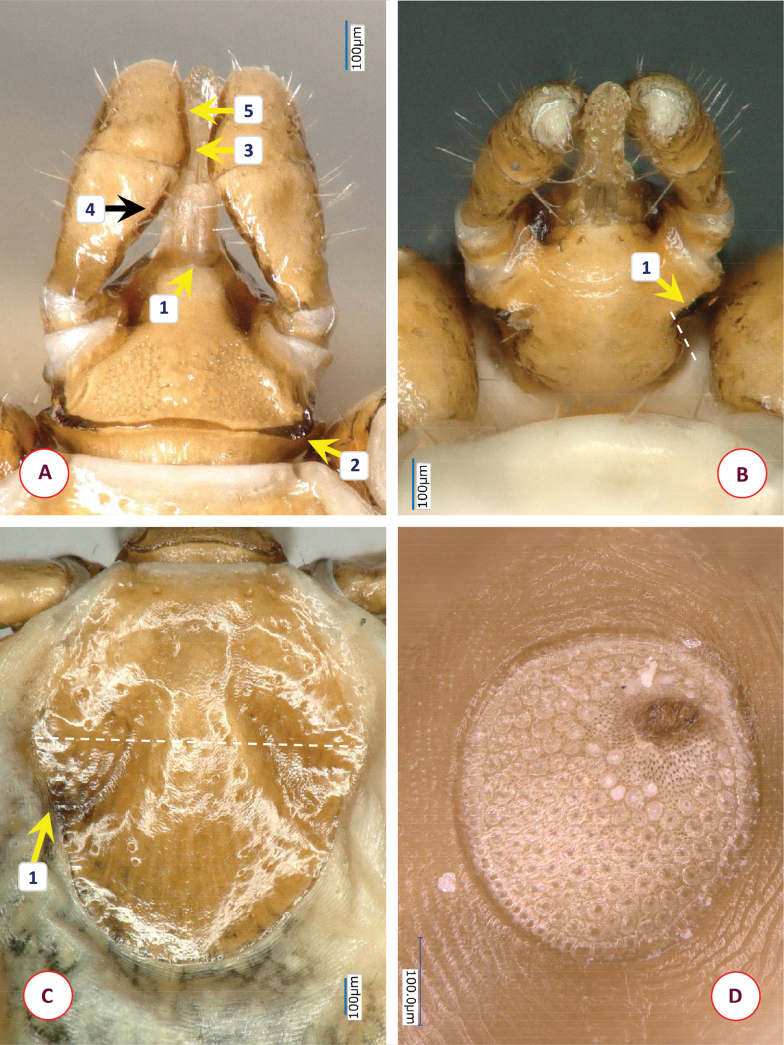
The morphology of *Ixodesfujitai* Hornok & Takano, sp. nov.: **A** dorsal aspect of basis capituli **B** ventral aspect of basis capituli **C** scutum **D** spiracular plate. Numbers are referred to in the text where relevant structures are described. Dashed lines indicate in **B** the posterolateral edge of ventral basis capituli and in **C** the maximum scutal width. Scale bars: 100 μm.

Scutum broad pentagonal (broadest slightly anterior to mid-length), anterior part trapezoid, posterolateral edge straight then rounded convex with wrinkled surface along its margin (Fig. 6C). Length of scutum 1.2, maximum width 1.03, ratio of length/width 1.17. Posterolateral, converging margin of scutum straight in ~ one 1/4 of scutal length. On the scutum curved, deep cervical grooves reaching posterolateral margin in its anterior part, i.e., at the end of straight edge (behind maximum width), with slight concavity (Fig. 6C–1). Punctuations moderately sparse, large. Dorsal scutal setae few in middle (length 0.015).

Alloscutum with sparse hair covering dorsally. Length of centrodorsal setae 0.125, idiosoma has sparse hair covering ventrally. Genital aperture between 2^nd^ and 3^rd^ intercoxal spaces, as broad horizontal C-shape. Genital groove posteriorly diverging, with narrowing at level of 4^th^ coxae. Spiracular plates subcircular, diameter 0.38, opening elongate (0.06), close to margin (Fig. 6D). Aeropyles in (2–3):(8–9) rows (minimum: maximum), densely packed, with margin broader than diameter of opening. Anal valves with two long (0.13) posterior setae. Anal groove slightly converging behind anus.

Length of gnathosoma (from palpal apices to posterior margin of basis capituli) 0.66, width of basis capituli dorsally 0.48. Ratio of gnathosomal length to basis capituli width 1.38. Basis capituli pentagonal (Fig. 6A), its sides anteriorly slightly then sharply converging. At the hypostomal basis dorsal flattening (plateau) (Fig. 6A–1), anteriorly gradually tapering to hypostomal base. Posterior margin wavy, slightly concave in middle, without cornuae but with squared, thickened corners (Fig. 6A–2). Areae porosae large, posterolaterally tapering, their interval 0.08. Ventrally basis capituli converging along straight, dark auricular ridges (Fig. 6B–1) to narrowing (“waist”), posteriorly diverging (Fig. 6B, dashed line).

Palps (dorsal view) short, club-shaped, broadest anterior to junction of segments II and III as a protuberance medially (Fig. 6A–3), anteriorly rounded. Edge as a broken line medially (both palpal segments II and III with relatively straight medial edge) (Fig. 6A–4, -5), straight laterally. Length 0.52, maximum width 0.19, ratio of length/width 2.7. Palpal hairs number 16–18 laterally (length 0.05–0.1), anteriorly five or six short (up to 0.05), medially few (0.02–0.05). Palpal segment II 0.29 long, palpal segment III 0.19 long, their ratio 1.5.

Hypostome broken on holotype (absent on paratype). Dental formula 2/2 where visible.

Legs long and slender. Coxa I subtriangular, coxae II and III trapezoidal with rounded medial edge (coxa II straight in mid-length), coxa IV semicircular. On coxae III and IV caudolateral long hair measuring 0.20–0.22. Tarsus I length 1.5, maximum diameter 0.17, length/diameter ratio 8.8. Hairs on tarsus I dorsally, laterally, and ventrally 0.07–0.30. Haller’s organ elongated, with grouped (non-linear) three anterior pit sensillae, one longer and stout (0.05).

##### Differential diagnosis.

**Figure 7.**
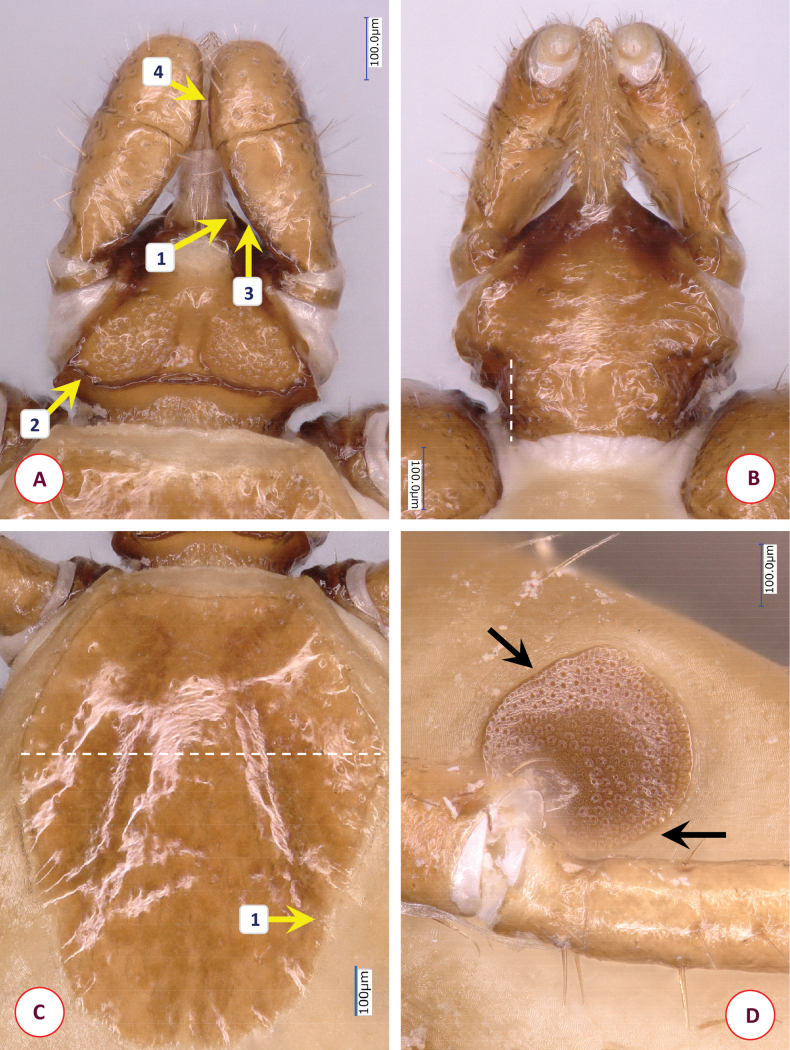
The morphology of *Ixodesariadnae*: **A** dorsal aspect of basis capituli **B** ventral aspect of basis capituli **C** scutum **D** spiracular plate. Numbers are referred to in the text where relevant structures are described. Dashed lines indicate in **B** the posterolateral edge of ventral basis capituli and in **C** the maximum scutal width. Scale bars: 100 μm.

*Ixodesfujitai* Hornok & Takano, sp. nov. can be easily distinguished from *I.simplex* based on its long legs (length to maximum diameter ratio above 8), and from members of the *I.vespertilionis* complex based on its short palps. Differences in comparison with females of the most similar species, *Ixodesariadnae* include the following characters of the latter. In *I.ariadnae* the scutum is slightly more elongated (ratio of length/width above 1.25 vs below 1.2 in *I.fujitai* Hornok & Takano, sp. nov.) (Fig. 7C). Posterolateral, converging margin of scutum in *I.ariadnae* straight in more than one third of scutal length (i.e., this part is longer than in *I.fujitai* Hornok & Takano, sp. nov.). On the scutum of *I.ariadnae* deep cervical grooves are straight in most of their length, reaching posterolateral margin in its middle, with more pronounced concavity (Fig. 7C–1). Punctuations on scutum very sparse. Spiracular plates of *I.ariadnae* subcircular but with straight portions of its edges (Fig. 7D), diameter smaller (0.33 vs 0.38 in *I.fujitai* Hornok & Takano, sp. nov.). Aeropyles loosely packed, with narrower margin than the diameter of their opening. Hypostome has laterally thickened basis (where sides are parallel with longitudinal axis) (Fig. 7A–1). Caudolateral corner of dorsal basis blunt, oblique (Fig. 7A–2) (vs squared, thickened angle in *I.fujitai* Hornok & Takano, sp. nov.). Posterior margin of basis wavy, strongly concave in middle (vs slightly in *I.fujitai* Hornok & Takano, sp. nov.). Ventrally basis capituli converging to narrow “waist” posterior to which it is nearly parallel (Fig. 7B dashed line); auricular ridge barely visible, scarcely sclerotised. In *I.ariadnae*, at the junction of palpal segments II and III no protuberance medially; their medial edge curved (Fig. 7A–3, -4) (vs straighter in *I.fujitai* Hornok & Takano, sp. nov.).

##### Gene sequences.

Complete mitochondrial genome sequence was deposited in the GenBank (LC769934). All accession numbers relevant to the new species are listed in Suppl. material 1.

##### Host record.

Known host species: *Murinahilgendorfi*.

##### Etymology.

The name of the new species refers to the collector of holotype, the late Dr. Hiromi Fujita (Ohara General Hospital), who was a leading researcher and major author on the topic of ticks and tick-borne pathogens.

## ﻿Discussion

Previously, when the molecular taxonomy of bat-specialist ticks was surveyed in Eurasia, the results indicated that several species new to science might exist among them in Asia (Hornok et al. 2015b). However, bat ticks are rare in Japan, in part because the percentage of parasitised bats is usually low (Yamauchi and Funakoshi 2000). Nevertheless, during the past years collections of bat ticks continued and in the present study females were examined both morphologically and molecularly, to describe three candidates as new species.

**Figure 8.**
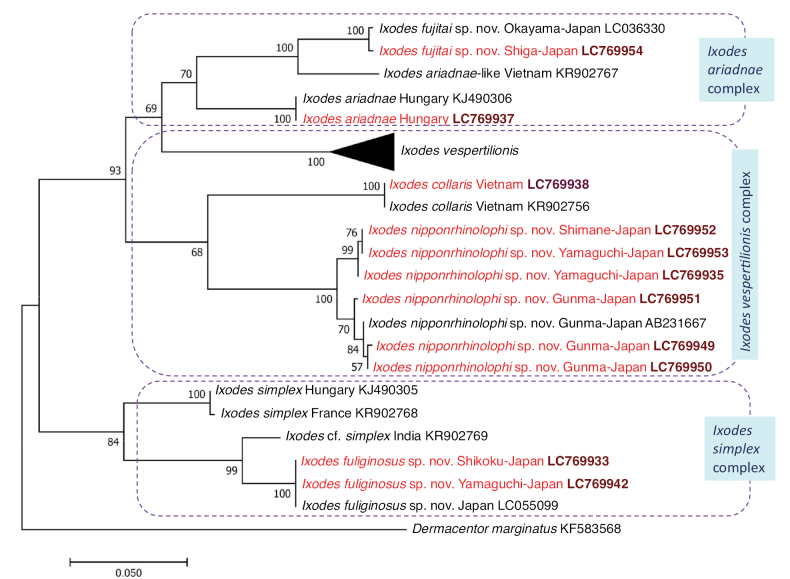
Maximum Likelihood phylogenetic tree of bat-associated ticks based on the coxI gene. In each row of individual sequences, the region/country of origin and the GenBank accession number are shown after the species name. Rows of sequences from this study are indicated with red fonts and bold accession numbers. Nineteen sequences of *I.vespertilionis* were used (JX394205–JX394208; KJ490307–KJ490311; KR902757–KR902777) but their clade is shown collapsed (as a triangle).

Considering molecular genetic comparison of ixodid ticks in general, the homologies of coxI sequences were reported to be above 93.9% within species, and below 94.4% between species (Lu et al. 2013). In line with this, the proposed average sequence divergence between closely related species (as a reference value delineating species) is 6.1% in the coxI, the so-called barcoding gene (Lv et al. 2014). This was considered when comparing the coxI sequences of each new species with its closest relative in a phylogenetic context (Fig. 8).

*Ixodesnipponrhinolophi* Hornok & Takano, sp. nov. was formerly considered *I.vespertilionis*. In the morphological illustration of the latter species collected in Japan (Yamaguti et al. 1971), certain morphologic characters were shown to be different from *I.vespertilionis* occurring in Europe (e.g., the arrangement of sensillae in Haller’s organ) and other characters as different from *I.collaris* described from Vietnam (e.g., the shape of scutum and the basis capituli ventrally). Phylogenetically, the closest relative of *I.nipponrhinolophi* Hornok & Takano, sp. nov. is *I.collaris* (Fig. 8). Based on coxI sequences, the genetic divergence between these two species is in the range of 11.9–12.5% (74–78/624 bp) which exceeds (doubles) the average limit of interspecific boundary, 6.1% (Lv et al. 2014). This, as well as the morphological differences described above, fully justify the taxonomical status of *I.nipponrhinolophi* Hornok & Takano, sp. nov. as a new species.

Regarding bat species, one of the most important hosts of *I.nipponrhinolophi* Hornok & Takano, sp. nov. in Japan is the Greater Horseshoe bat, *Rhinolophusferrumequinum* (Arthur 1956; Yamaguti et al. 1971). This species is widely distributed across Eurasia, from south-western Europe to Japan (Frutos et al. 2021). By contrast, the preferred host species of *I.collaris*, the Intermediate Horseshoe bat, *R.affinis* Horsfield, 1823 (Hornok et al. 2019) occurs only in Southeast Asia (Frutos et al. 2021).

Moreover, within *I.nipponrhinolophi* Hornok & Takano, sp. nov., the coxI sequence identity between specimens collected in southwestern Japan (Yamaguchi prefecture) and central Japan (Gunma prefecture) is very similar in magnitude (98%) to the difference reported between *I.vespertilionis* from Western Europe (France) and Central Europe (Hungary). In both cases there is a geographical barrier to bat migration and no direct contact between relevant bat populations, i.e., the Alps in Europe and the Alps in Japan, providing the most likely explanation for this genetic difference and reproductive isolation behind this phenomenon.

Long-known records and illustrations of *I.vespertilionis* or closely related species from various regions allow the conclusion on its occurrence in Asia. Thus, based on the morphology of the scutum and basis capituli, *I.vespertilionis* (and not *I.collaris* or *I.nipponrhinolophi* Hornok & Takano, sp. nov.) was reported from Russia (Pomerantzev 1950) and China (Kuofan and Zaijie 1991).

*Ixodesfuliginosus* Hornok & Takano, sp. nov. was formerly considered *I.simplex*, with several similar and different characters noted above or shown between specimens from Europe and Eastern Asia: for instance, the anal groove was reported to be pointed in front of the anus in case of both *I.simplex* (Arthur 1956) and its variant in Japan (Yamaguti et al. 1971), so this is not part of their differential diagnosis. On the other hand, the front of basis capituli and areae porosae were reported to be important distinguishing characters between *Pholeoixodes* species (Hornok et al. 2017), which (according to the taxonomical requirement of monophyly) should include *Eschatocephalus*, thus *I.simplex* and *I.fuliginosus* Hornok & Takano, sp. nov. (Hornok et al. 2023). Relevant differences between *I.simplex* from Europe and Japan were already noted by Arthur (1956), i.e., focusing on the tectum, and the shapes of the basis capituli and areae porosae. Similarly, the number of posterior setae (hairs) is fewer, 8–12 on coxae IV in case of *I.simplex*, also shown or stated in previous descriptions and illustrations of this species (Babos and Janisch 1958; Hornok 2017), in contrast to the higher number (16–18) in *I.fuliginosus* Hornok & Takano, sp. nov.

Phylogenetically, the closest relative of *I.fuliginosus* sp. nov. is *I.simplex* (Fig. 8). Based on coxI sequences, the genetic divergence between these two species is 10–10.6% (60/601–66/623 bp) which exceeds (almost doubles) the average limit of interspecific boundary, 6.1% (Lv et al. 2014), thus confirming the taxonomical status of *I.fuliginosus* sp. nov. as a new species. The taxonomy of the preferred (specific) host of *I.simplex*, i.e., the Common Bent-wing bat, *Miniopterusschreibersii* (Kuhl, 1817) was revised, because previously it was thought to be a uniform species across Eurasia/Old World (Li et al. 2015). Although in this study *I.fuliginosus* Hornok & Takano, sp. nov. was more often collected from Big-footed myotis, *Myotismacrodactylus*, its main host is probably the Eastern Bent-wing bat, *M.fuliginosus* (as reported in Hornok et al. 2015b) which is distributed from the Caucasus to southern Asia including China, the Korean Peninsula, and Japan (Li et al. 2015). In line with this, the relatively low (~ 4%) coxI sequence difference of *I.fuliginosus* Hornok & Takano, sp. nov. from *I.simplex* reported from India (KR902774: Hornok et al. 2015b) suggests that *Ixodesfuliginosus* Hornok & Takano, sp. nov. has a broad geographical range in southern Asia.

*Ixodesfujitai* Hornok & Takano, sp. nov. is most similar morphologically to *I.ariadnae*, and phylogenetically belongs to a sister group of its specimens from Europe (Fig. 8). Based on coxI sequences, the genetic divergence between these two species is 10.3% (65/630 bp) which significantly exceeds the average limit of interspecific boundary, 6.1% (Lv et al. 2014), further supporting the taxonomical status of *Ixodesfujitai* Hornok & Takano, sp. nov. as a new species.

Importantly, Hilgendorf’s Tube-nosed bat, *Murinahilgendorfi* (Murininae), the only hitherto known bat host of *I.fujitai* Hornok & Takano, sp. nov. (Hornok et al. 2015b; this study) is closely related to *Myotis* species (Ruedi et al. 2013), the preferred hosts of *I.ariadnae* (Hornok et al. 2014, 2016).

The results of phylogenetic analyses also justify that bat-associated ixodid tick species form complexes based on their general morphology, i.e., members of the *I.ariadnae* complex have short palps and long legs, those in the *I.vespertilionis* complex have long palps and long legs, whereas species of the *I.simplex* complex have short palps and short legs (Fig. 8). Based on the coxI gene, the *I.ariadnae* and the *I.simplex* complexes are monophyletic.

In conclusion, in the case of all three bat-associated tick species that are newly described in this study, the coxI sequence differences from formerly known members of their species complexes strongly support their status as separate species. In addition, previous data on the host preference of these groups were also confirmed, i.e., members of *I.ariadnae* complex were collected from vesper bats (Vespertilionidae), those in the *I.vespertilionis* complex mostly infest horseshoe bats (Rhinolophidae), and species of the *I.simplex* complex typically associate with bent-winged bats, Miniopteridae (Hornok et al. 2016). However, larger scale sampling will be necessary in the future to draw final conclusions in this respect.

As illustrated above, new ixodid tick species might exist and await discovery and description even in such exhaustively explored countries as Japan. Although ixodid bat ticks are relatively rare in the region of eastern Asia, further steps need to be taken to find and describe males and immature stages of these new species, as well as to ascertain their more complete geographical and host ranges.

## Supplementary Material

XML Treatment for Ixodes nipponrhinolophi

XML Treatment for Ixodes fuliginosus

XML Treatment for Ixodes fujitai

## ﻿Additional information

### Author ORCIDs

Ai Takano https://orcid.org/0000-0002-5919-3738

Mamoru Takahashi https://orcid.org/0000-0003-1511-3756

Jenő Kontschán https://orcid.org/0000-0001-8274-4238

Sándor Hornok https://orcid.org/0000-0002-1125-5178

### Data availability

All of the data that support the findings of this study are available in the main text or Supplementary Information.

## Supplementary materials

Supplementary material 1 Collection data and accession numbers of specimens Data type pdf https://binary.pensoft.net/file/906292This dataset is made available under the Open Database License (http://opendatacommons.org/licenses/odbl/1.0/). The Open Database License (ODbL) is a license agreement intended to allow users to freely share, modify, and use this Dataset while maintaining this same freedom for others, provided that the original source and author(s) are credited.Ai Takano, Takeo Yamauchi, Mamoru Takahashi, Hiroshi Shimoda, Yasuhiro Gotoh, Junko Mizuno, Michio Natsume, Jenő Kontschán, Dávid Kováts, Vuong Tan Tu, Sándor Hornok

Supplementary material 2 Additional morphological details of *Ixodesnipponrhinolophi* Hornok & Takano, sp. nov Data type pdf Explanation note (**A**) dorsal habitus, (**B**) ventral habitus, (**C**) spiracular plate, (**D**) anal valves. Scale bars: 500 μm (**A, B**); 100 μm (**C, D**). https://binary.pensoft.net/file/906293This dataset is made available under the Open Database License (http://opendatacommons.org/licenses/odbl/1.0/). The Open Database License (ODbL) is a license agreement intended to allow users to freely share, modify, and use this Dataset while maintaining this same freedom for others, provided that the original source and author(s) are credited.Ai Takano, Takeo Yamauchi, Mamoru Takahashi, Hiroshi Shimoda, Yasuhiro Gotoh, Junko Mizuno, Michio Natsume, Jenő Kontschán, Dávid Kováts, Vuong Tan Tu, Sándor Hornok

Supplementary material 3 Additional morphological details of *Ixodescollaris* Data type pdf Explanation note (**A**) dorsal habitus, (**B**) ventral habitus, (**C**) spiracular plate, (**D**) anal valves. Scale bars: 1000 μm (**A, B**); 100 μm (**C, D**). https://binary.pensoft.net/file/906294This dataset is made available under the Open Database License (http://opendatacommons.org/licenses/odbl/1.0/). The Open Database License (ODbL) is a license agreement intended to allow users to freely share, modify, and use this Dataset while maintaining this same freedom for others, provided that the original source and author(s) are credited.Ai Takano, Takeo Yamauchi, Mamoru Takahashi, Hiroshi Shimoda, Yasuhiro Gotoh, Junko Mizuno, Michio Natsume, Jenő Kontschán, Dávid Kováts, Vuong Tan Tu, Sándor Hornok

Supplementary material 4 Additional morphological details of *Ixodesfuliginosus* Hornok & Takano, sp. nov. Data type pdf Explanation note (**A**) dorsal habitus, (**B**) ventral habitus, (**C**) spiracular plate of holotype, (**D**) paratype. Numbers are referred to in the text where relevant structures are described. Scale bars: 100 μm. https://binary.pensoft.net/file/906295This dataset is made available under the Open Database License (http://opendatacommons.org/licenses/odbl/1.0/). The Open Database License (ODbL) is a license agreement intended to allow users to freely share, modify, and use this Dataset while maintaining this same freedom for others, provided that the original source and author(s) are credited.Ai Takano, Takeo Yamauchi, Mamoru Takahashi, Hiroshi Shimoda, Yasuhiro Gotoh, Junko Mizuno, Michio Natsume, Jenő Kontschán, Dávid Kováts, Vuong Tan Tu, Sándor Hornok

Supplementary material 5 Additional morphological details of *Ixodessimplex* Data type pdf Explanation note (**A**) dorsal habitus, (**B**) ventral habitus, (**C**) spiracular plate, (**D**) anal valves, anal groove. Numbers are referred to in the text where relevant structures are described. Scale bars: 100 μm. https://binary.pensoft.net/file/906296This dataset is made available under the Open Database License (http://opendatacommons.org/licenses/odbl/1.0/). The Open Database License (ODbL) is a license agreement intended to allow users to freely share, modify, and use this Dataset while maintaining this same freedom for others, provided that the original source and author(s) are credited.Ai Takano, Takeo Yamauchi, Mamoru Takahashi, Hiroshi Shimoda, Yasuhiro Gotoh, Junko Mizuno, Michio Natsume, Jenő Kontschán, Dávid Kováts, Vuong Tan Tu, Sándor Hornok

## References

[B1] Arthur DR (1956) The *Ixodes* ticks of Chiroptera (Ixodoidea, Ixodidae). The Journal of Parasitology 42(2): 180–196. 10.2307/3274734 13320260

[B2] Babos A Janisch N (1958) *Ixodeschiropterorum* sp. n., eine neue Zeckenart in Ungarn. Acta Veterinaria Academiae Scientiarum Hungaricae 8: 389–399. 10.1007/BF03156912

[B3] Beati L Keirans JE (2001) Analysis of the systematic relationships among ticks of the genera *Rhipicephalus* and *Boophilus* (Acari: Ixodidae) based on mitochondrial 12S ribosomal DNA gene sequences and morphological characters. The Journal of Parasitology 87(1): 32–48. 10.1645/0022-3395(2001)087[0032:AOTSRA]2.0.CO;2 11227901

[B4] Beltz LA (2018) Bats and human health, Ebola, SARS, Rabies and Beyond. Wiley, Hoboken, Nj, USA, 402 pp. 10.1002/9781119150060

[B5] Brook CE Dobson AP (2015) Bats as ‘special’ reservoirs for emerging zoonotic pathogens. Trends in Microbiology 23(3): 172–180. 10.1016/j.tim.2014.12.004 25572882 PMC7126622

[B6] Folmer O Black M Hoeh W Lutz R Vrijenhoek R (1994) DNA primers for amplification of mitochondrial cytochrome C oxidase subunit I from diverse metazoan invertebrates. Molecular Marine Biology and Biotechnology 3: 294–299. 7881515

[B7] Frutos R Serra-Cobo J Pinault L Lopez Roig M Devaux CA (2021) Emergence of bat-related betacoronaviruses: Hazard and Risks. Frontiers in Microbiology 12: 591535. 10.3389/fmicb.2021.591535 PMC8005542 33790874

[B8] Han H-J Wen H Zhou C-M Chen F-F Luo L-M Liu J Yu X-J (2015) Bats as reservoirs of severe emerging infectious diseases. Virus Research 205: 1–6. 10.1016/j.virusres.2015.05.006 25997928 PMC7132474

[B9] Hornok S (2017) *Ixodessimplex* Neumann, 1906. In: Estrada-Peña A Mihalca AD Petney TN (Eds) Ticks of Europe and North Africa: A guide to species identification.Springer International Publishing, Cham, 103–107. 10.1007/978-3-319-63760-0_21

[B10] Hornok S Kontschán J Kováts D Kovács R Angyal D Görföl T Polacsek Z Kalmár Z Mihalca AD (2014) Bat ticks revisited: *Ixodesariadnae* sp. nov. and allopatric genotypes of *I.vespertilionis* in caves of Hungary. Parasites & Vectors 7(1): 202. 10.1186/1756-3305-7-202 24766822 PMC4029976

[B11] Hornok S Estók P Kováts D Flaisz B Takács N Szőke K Krawczyk A Kontschán J Gyuranecz M Fedák A Farkas R Haarsma AJ Sprong H (2015a) Screening of bat faeces for arthropod-borne apicomplexan protozoa: *Babesiacanis* and *Besnoitiabesnoiti*-like sequences from Chiroptera. Parasites & Vectors 8(1): 441. 10.1186/s13071-015-1052-6 26315069 PMC4552134

[B12] Hornok S Estrada-Peña A Kontschán J Plantard O Kunz B Mihalca AD Thabah A Tomanović S Burazerović J Takács N Görföl T Estók P Tu VT Szőke K Fernández de Mera IG de la Fuente J Takahashi M Yamauchi T Takano A (2015b) High degree of mitochondrial gene heterogeneity in the bat tick species *Ixodesvespertilionis*, *I.ariadnae* and *I.simplex* from Eurasia. Parasites & Vectors 8(1): 457. 10.1186/s13071-015-1056-2 26382218 PMC4573304

[B13] Hornok S Szőke K Kováts D Estók P Görföl T Boldogh SA Takács N Kontschán J Földvári G Barti L Corduneanu A Sándor AD (2016) DNA of piroplasms of ruminants and dogs in ixodid bat ticks. PLoS ONE 11(12): e0167735. 10.1371/journal.pone.0167735 PMC5145180 27930692

[B14] Hornok S Sándor AD Beck R Farkas R Beati L Kontschán J Takács N Földvári G Silaghi C Meyer-Kayser E Hodžić A Tomanović S Abdullah S Wall R Estrada-Peña A Duscher GG Plantard O (2017) Contributions to the phylogeny of Ixodes (Pholeoixodes) canisuga, I. (Ph.) kaiseri, I. (Ph.) hexagonus and a simple pictorial key for the identification of their females. Parasites & Vectors 10(1): 545. 10.1186/s13071-017-2424-x 29100530 PMC5670724

[B15] Hornok S Murányi D Kontschán J Tu VT (2019) Description of the male and the larva of *Ixodescollaris* Hornok, 2016 with drawings of all stages. Parasites & Vectors 12(1): 144. 10.1186/s13071-019-3365-3 30914054 PMC6434778

[B16] Hornok S Mihalca AD Kontschán J Takács N Fedorov D Plantard O Sándor AD (2023) Phylogenetic analyses of *Ixodesrugicollis* with notes on its morphology in comparison with *Ixodescornutus*. Parasites & Vectors 16(1): 106. 10.1186/s13071-023-05718-z 36927655 PMC10022209

[B17] Hutterer R Ivanova T Meyer-Cords C Rodrigues L (2005) Bat migrations in Europe. A review of banding data and literature. Naturschutz und Biologische Viefalt 28. Federal Agency for Nature Conservation, Bonn, 162 pp.

[B18] Jaenson TG Tälleklint L Lundqvist L Olsen B Chirico J Mejlon H (1994) Geographical distribution, host associations, and vector roles of ticks (Acari: Ixodidae, Argasidae) in Sweden. Journal of Medical Entomology 31(2): 240–256. 10.1093/jmedent/31.2.240 8189415 PMC7107449

[B19] Jongejan F Uilenberg G (2004) The global importance of ticks. Parasitology 129(S1, suppl): S3–S14. 10.1017/S0031182004005967 15938502

[B20] Kajitani R Yoshimura D Ogura Y Gotoh Y Hayashi T Itoh T (2020) Platanus B: an accurate *de novo* assembler for bacterial genomes using an iterative error-removal process. DNA Research 27(3): dsaa014. 10.1093/dnares/dsaa014 PMC7433917 32658266

[B21] Kelava S Mans BJ Shao R Moustafa MAM Matsuno K Takano A Kawabata H Sato K Fujita H Ze C Plantard O Hornok S Gao S Barker D Barker SC Nakao R (2021) Phylogenies from mitochondrial genomes of 120 species of ticks: Insights into the evolution of the families of ticks and of the genus *Amblyomma*. Ticks and Tick-Borne Diseases 12(1): 101577. 10.1016/j.ttbdis.2020.101577 33120251

[B22] Klimpel S Mehlhorn H (2014) Bats (Chiroptera) as vectors of diseases and parasites: facts and myths. Springer Publications, New York, 187 pp. 10.1007/978-3-642-39333-4

[B23] Kumar S Stecher G Tamura K (2016) MEGA7: Molecular Evolutionary Genetics Analysis Version 7.0 for Bigger Datasets. Molecular Biology and Evolution 33(7): 1870–1874. 10.1093/molbev/msw054 27004904 PMC8210823

[B24] Kuofan T Zaijie J (1991) Acari: Ixodidae. Economic Insect Fauna of China. Science Press, Beijing, Fasc 39: 72–76.

[B25] Li S Sun K Lu G Lin A Jiang T Jin L Hoyt JR Feng J (2015) Mitochondrial genetic differentiation and morphological difference of *Miniopterusfuliginosus* and *Miniopterusmagnater* in China and Vietnam. Ecology and Evolution 5(6): 1214–1223. 10.1002/ece3.1428 25859327 PMC4377265

[B26] Lu X Lin XD Wang JB Qin XC Tian JH Guo WP Fan FN Shao R Xu J Zhang YZ (2013) Molecular survey of hard ticks in endemic areas of tick-borne diseases in China. Ticks and Tick-Borne Diseases 4(4): 288–296. 10.1016/j.ttbdis.2013.01.003 23538111

[B27] Lv J Wu S Zhang Y Chen Y Feng C Yuan X Jia G Deng J Wang C Wang Q Mei L Lin X (2014) Assessment of four DNA fragments (COI, 16S rDNA, ITS2, 12S rDNA) for species identification of the Ixodida (Acari: Ixodida). Parasites & Vectors 7(1): 93. 10.1186/1756-3305-7-93 24589289 PMC3945964

[B28] Norris DE Klompen JSH Black WC (1999) Comparison of the mitochondrial 12S and 16S ribosomal DNA genes in resolving phylogenetic relationships among hard ticks (Acari: Ixodidae). Annals of the Entomological Society of America 92(1): 117–129. 10.1093/aesa/92.1.117

[B29] Oba M Omatsu T Takano A Fujita H Sato K Nakamoto A Takahashi M Takada N Kawabata H Ando S Mizutani T (2016) A novel Bunyavirus from the soft tick, *Argasvespertilionis*, in Japan. The Journal of Veterinary Medical Science 78(3): 443–445. 10.1292/jvms.15-0536 26498534 PMC4829514

[B30] Péter Á Barti L Corduneanu A Hornok S Mihalca AD Sándor AD (2021) First record of *Ixodessimplex* found on a human host, with a review of cases of human infestation by bat tick species occurring in Europe. Ticks and Tick-Borne Diseases 12(4): 101722. 10.1016/j.ttbdis.2021.101722 33865178

[B31] Piksa K Nowak-Chmura M Siuda K (2013) First case of human infestation by the tick *Ixodesvespertilionis* (Acari: Ixodidae). International Journal of Acarology 38(1): 1–2. 10.1080/01647954.2012.737831

[B32] Plowright RK Foley P Field HE Dobson AP Foley JE Eby P Daszak P (2011) Urban habituation, ecological connectivity and epidemic dampening: The emergence of Hendra virus from flying foxes (*Pteropus* spp.). Proceedings of the Royal Society B, Biological Sciences 278(1725): 3703–3712. 10.1098/rspb.2011.0522 PMC3203503 21561971

[B33] Pomerantzev BI (1950) Ixodid ticks (Ixodidae). Fauna of U.S.S.R., Arachnida. The American Institute of Biological Sciences, Washington 4(2): 67–70.

[B34] Ruedi M Stadelmann B Gager Y Douzery EJ Francis CM Lin LK Guillén-Servent A Cibois A (2013) Molecular phylogenetic reconstructions identify East Asia as the cradle for the evolution of the cosmopolitan genus *Myotis* (Mammalia, Chiroptera). Molecular Phylogenetics and Evolution 69(3): 437–449. 10.1016/j.ympev.2013.08.011 23988307

[B35] Simmons NB (2005) Order Chiroptera. In: Wilson DE Reeder DM (Eds) Mammal species of the world: a taxonomic and geographic reference.Baltimore, Johns Hopkins University Press, vol. 1, 312–529.

[B36] Simmons NB Cirranello AL (2023) Bat Species of the World: A taxonomic and geographic database. https://batnames.org/ [Accessed on 03/16/2023]

[B37] Ushijima Y Oliver Jr JH Keirans JE Tsurumi M Kawabata H Watanabe H Fukunaga M (2003) Mitochondrial sequence variation in *Carioscapensis* (Neumann), a parasite of seabirds, collected on Torishima Island in Japan. The Journal of Parasitology 89(1): 196–198. 10.1645/0022-3395(2003)089[0196:MSVICC]2.0.CO;2 12659332

[B38] Yamaguti N Tipton VJ Keegan HL Toshioka S (1971) Ticks in Japan, Korea and Ryukyu Islands. Brigham Young University Science Bulletin, Biological Series 15: 1–226. 10.5962/bhl.part.25691

[B39] Yamauchi T Funakoshi K (2000) Ticks from Chiroptera (Mammalia) of the mainland Kyushu, Japan (Acari: Ixodoidea). Journal of the Acarological Society of Japan 9: 51–54. 10.2300/acari.9.51

