# Supplementary material for: ﻿Description of three new bat-associated species of hard ticks (Acari, Ixodidae) from Japan
Source: Zookeys. 2023 Sep 15;1180:1–26. doi: 10.3897/zookeys.1180.108418 (PMC10517414; doi:10.3897/zookeys.1180.108418)
Supplement: Supplementary material 1 — Collection data and accession numbers of specimens [file zookeys-1180-001_article-108418__-s001.pdf]

Supplementary Table 1. Collection data and accession numbers of specimens.

	Tick species	Stage	Source of origin	Sample code	Collected by	Country of collection	Data collected: yy-mm-dd	Morphological investigation	Accession Number COI	Accession Number 16S	Accession Number 12S	Accession Number mt-genome	Google map GPS
I.vespertilionis -like	*Ixodes nipponrhinolophi*	Female (molted)	*Rhinolophus cornutus*	YB47	A. Takano	Japan (Yamaguchi)	2021.02.25	yes	ND	ND	ND	ND	34.251084, 131.243056
	Ixodes nipponrhinolophi	Female	*Rhinolophus ferrumequinum*		K. Funakoshi	Japan (Kumamoto)	1973.12.24	yes	ND	ND	ND	ND	32.252183, 130.651239
	Ixodes nipponrhinolophi	Female	*Rhinolophus ferrumequinum*		K. Funakoshi	Japan (Kumamoto)	1979.04.12	yes	ND	ND	ND	ND	32.252183, 130.651239
	Ixodes nipponrhinolophi	Female	*Rhinolophus ferrumequinum*		K. Funakoshi	Japan (Kumamoto)	1979.05.10	yes	ND	ND	ND	ND	32.252183, 130.651239
	Ixodes nipponrhinolophi	Female	*Rhinolophus ferrumequinum*		K. Funakoshi	Japan (Kumamoto)	1979.05.25	yes	ND	ND	ND	ND	32.252183, 130.651239
	Ixodes nipponrhinolophi	Female	*Rhinolophus cornutus*	4673	M. Takahashi	Japan (Gunma)	2005.04.23	yes	ND	ND	ND	ND	36.086915, 138.721945
	Ixodes nipponrhinolophi	Male	Cave wall		S. Ando	Japan (Shimane)	2020.06.04	ND	LC769952	LC769948	LC769945	ND	
	Ixodes nipponrhinolophi	Larva	*Rhinolophus ferrumequinum*	6157	M. Takahashi	Japan (Gunma)	2015.05.12	ND	LC769949	LC769946	LC769943	ND	
	Ixodes nipponrhinolophi	Larva	*Rhinolophus cornutus*	6169	M. Takahashi	Japan (Gunma)	2015.06.02	ND	LC769950	+*	+*	ND	
	Ixodes nipponrhinolophi	Larva	*Rhinolophus cornutus*	6175	M. Takahashi	Japan (Gunma)	2015.06.02	ND	LC769951	LC769947	LC769944	ND	
	Ixodes nipponrhinolophi	Larva	*Rhinolophus cornutus*	YB46-1	A. Takano	Japan (Yamaguchi)	2021.02.25	ND	LC769953	ND	ND	ND	
	Ixodes nipponrhinolophi	Larva	*Rhinolophus cornutus*	YB48	A. Takano	Japan (Yamaguchi)	2021.02.25	ND	-	-	-	LC769935	
	Ixodes vespertilionis	Female	Pálvölgyi Cave (wall)	KD36	D. Kováts	Hungary (Budapest)	2018.12.01	yes	ND	ND	ND	ND	
	Ixodes vespertilionis	Female	Leány Cave (wall)	CV1	D. Kováts	Hungary (Pilis Mountains)	2016.03.06	yes	ND	ND	ND	ND	
	Ixodes vespertilionis	Female	Leány Cave (wall)	CV142	D. Kováts	Hungary (Pilis Mountains)	2017.03.19	yes	ND	ND	ND	ND	
	Ixodes vespertilionis	Female	Leány Cave (wall)	CV143	D. Kováts	Hungary (Pilis Mountains)	2017.03.19	ND	LC769936	ND	ND	ND	
	Ixodes collaris	Female	*Hipposideros pomona*	VN14-0011	V. Tan Tu	Vietnam (Kon Tum)	2014.09.22	yes	ND	ND	ND	ND	
	Ixodes collaris	Nymph	*Rhinolophus affinis*	VN16-53	V. Tan Tu	Vietnam	2016.06.01	ND	LC769938	ND	ND	ND	
I.simplex -like	*Ixodes fuliginosus*	Female	*Myotis macrodactylus*	Kana2020	F. Sato	Japan (Kanagawa)	2022.09.30	yes	ND	LC769940	ND	ND	35.623170, 139.165542
	Ixodes fuliginosus	Female	*Miniopterus fuliginosus*		K. Funakoshi	Japan (Kumamoto)	1979.04.12	yes	ND	ND	ND	ND	32.252183, 130.651239
	Ixodes fuliginosus	Female	*Myotis macrodactylus*		K. Funakoshi	Japan (Oita)	1997.07.17	yes	ND	ND	ND	ND	33.228090, 130.981712
	Ixodes fuliginosus	Female	*Myotis macrodactylus*	5997	M. Takahashi	Japan (Gunma)	2014.06.17	yes	ND	+**	ND	ND	36.685602, 138.925637
	Ixodes fuliginosus	Nymph	*Myotis macrodactylus*	5995	M. Takahashi	Japan (Gunma)	2014.06.17	ND	ND	LC769939	ND	ND	
	Ixodes fuliginosus	Nymph	*Miniopterus fuliginosus*	YB14	A. Takano	Japan (Yamaguchi)	2021.02.15	ND	LC769942	LC769941	ND	ND	
	Ixodes fuliginosus	Male	*Murina ussuriensis*	Is_2020	R. Kuwata	Japan (Kochi)	2020.09.15	ND	-	-	-	LC769933	
	Ixodes simplex	Female	*Miniopterus schreibersii*		A. D. Sándor	Romania (Báziás)	2022.09.20-23.	yes	ND	ND	ND	ND	
I.ariadnae -like	*Ixodes fujitai*	Female	unknown		H. Fujita	Japan (Shiga)	1990.12.09	yes	ND	ND	ND	ND	35.222448, 136.291747
	Ixodes fujitai	Female	*Murina hilgendorfi*	Iv_Oka2013	M. Yamada	Japan (Okayama)	2013.03.09	yes	-	-	-	LC769934	34.961817, 133.631483
	Ixodes fujitai	Female	*Murina hilgendorfi*	Shiga2016	K. Okumura	Japan (Shiga)	2016.04.22	yes	LC769954	LC769955	LC769956	ND	35.222448, 136.291747
	Ixodes ariadnae	Female	Legény Cave (wall)	CV86	D. Kováts	Hungary (Pilis Mountains)	2017.03.05	yes	LC769937	ND	ND	ND	

ND = Not Done

*Sequence data 100% identical with that of 6157

**Sequence data 100% identical with that of 5995
