# Supplementary figures and images for: ﻿Description of three new bat-associated species of hard ticks (Acari, Ixodidae) from Japan
Source: Zookeys. 2023 Sep 15;1180:1–26. doi: 10.3897/zookeys.1180.108418 (PMC10517414; doi:10.3897/zookeys.1180.108418)

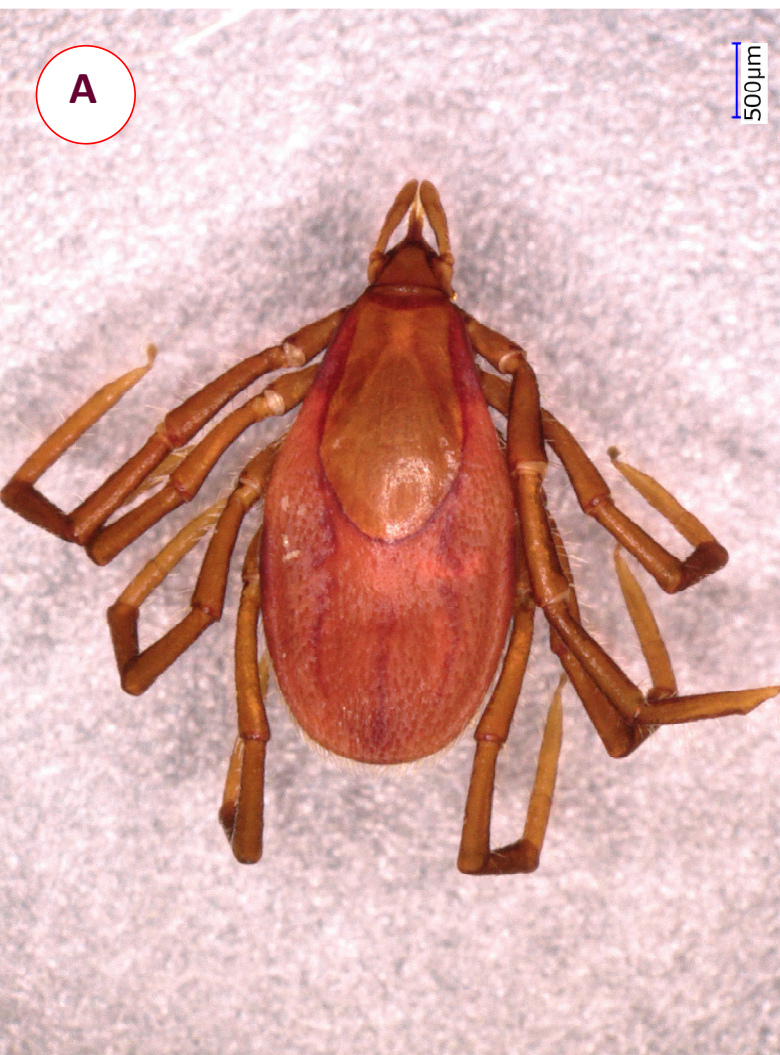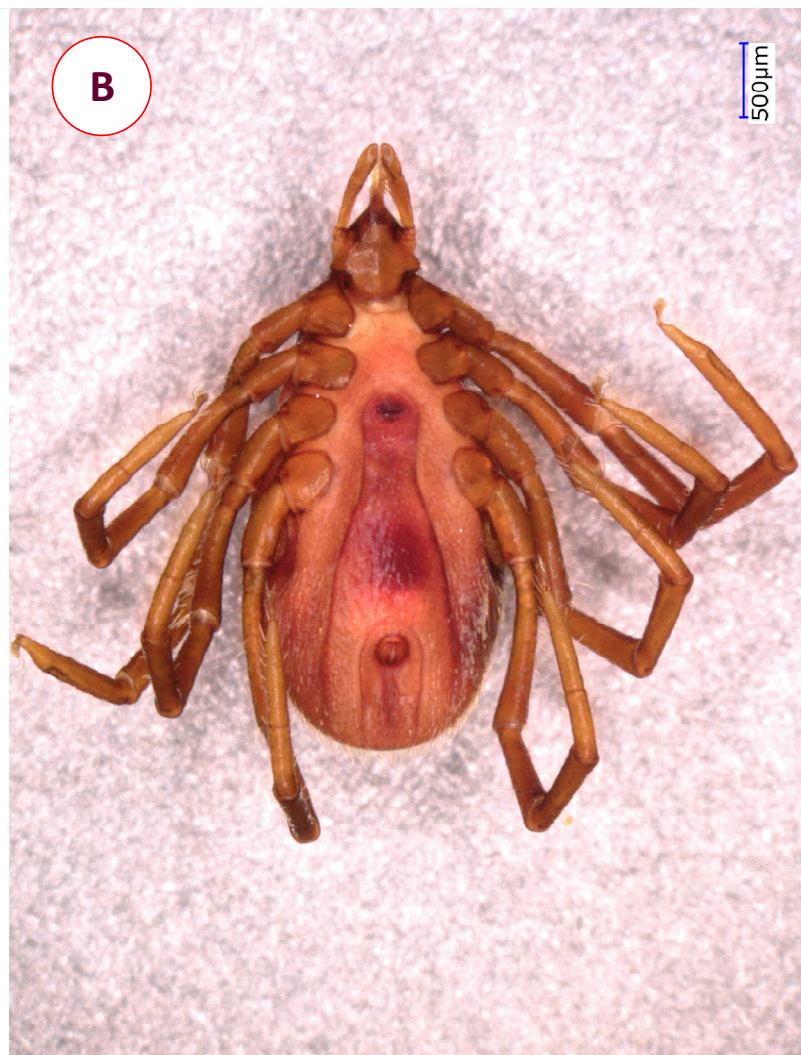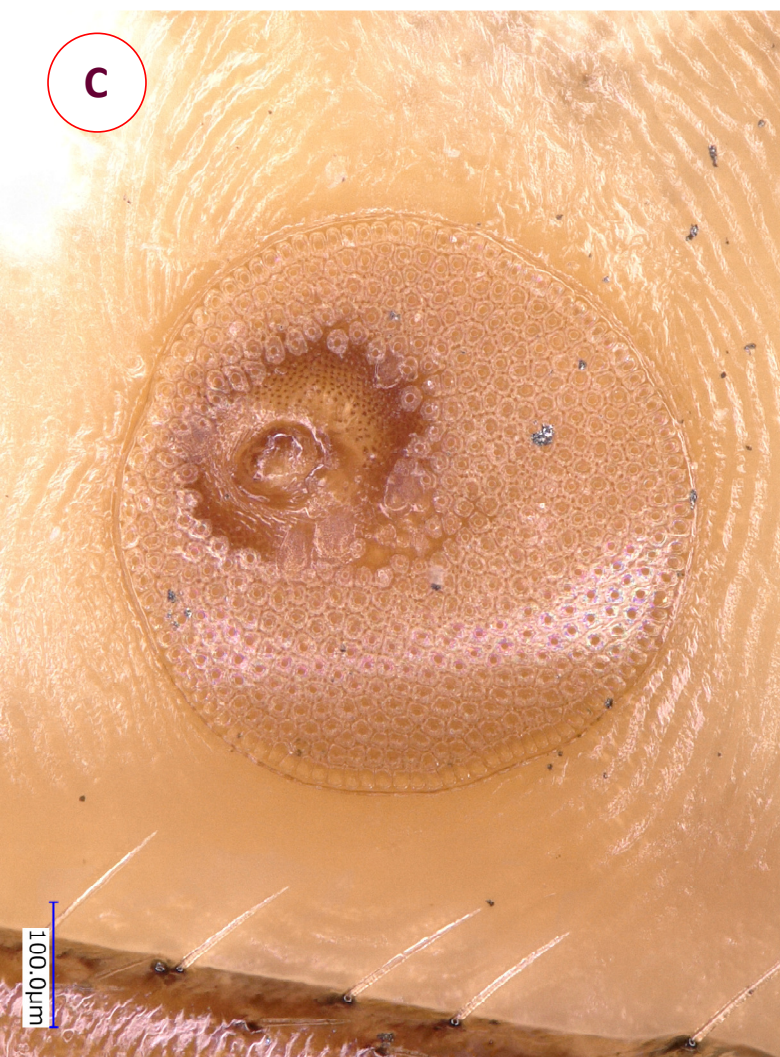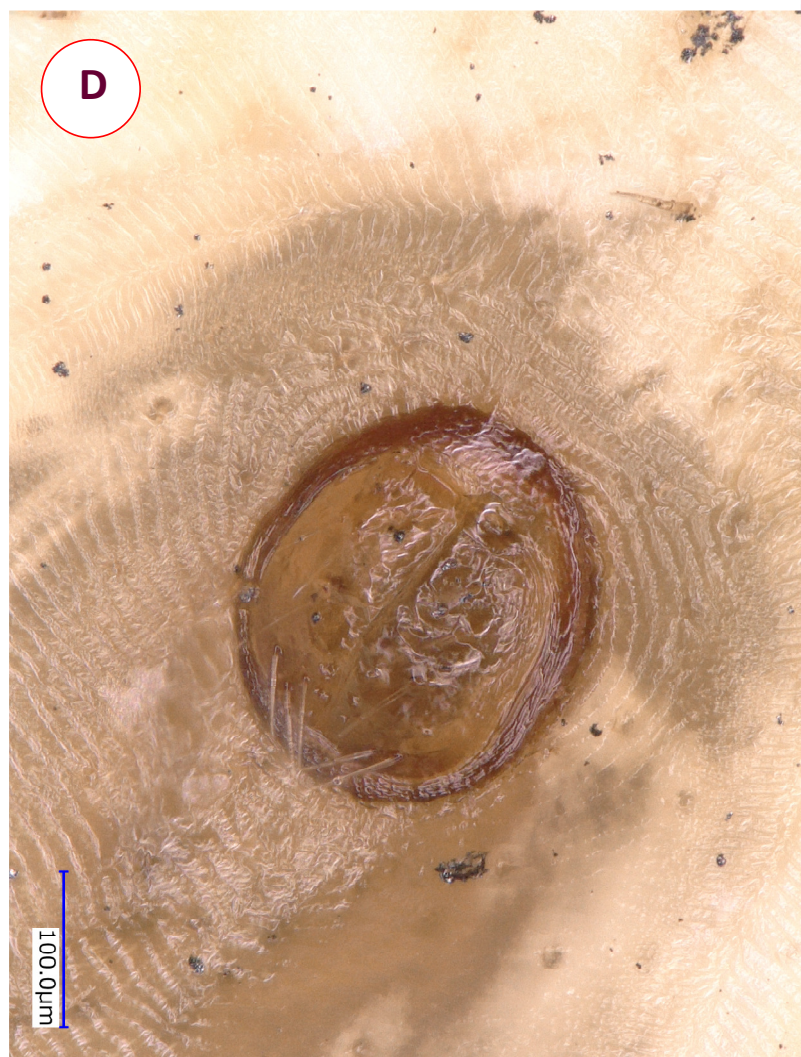

Supplement: Supplementary material 2 — Additional morphological details of Ixodesnipponrhinolophi Hornok & Takano, sp. nov [file zookeys-1180-001_article-108418__-s002.pdf]

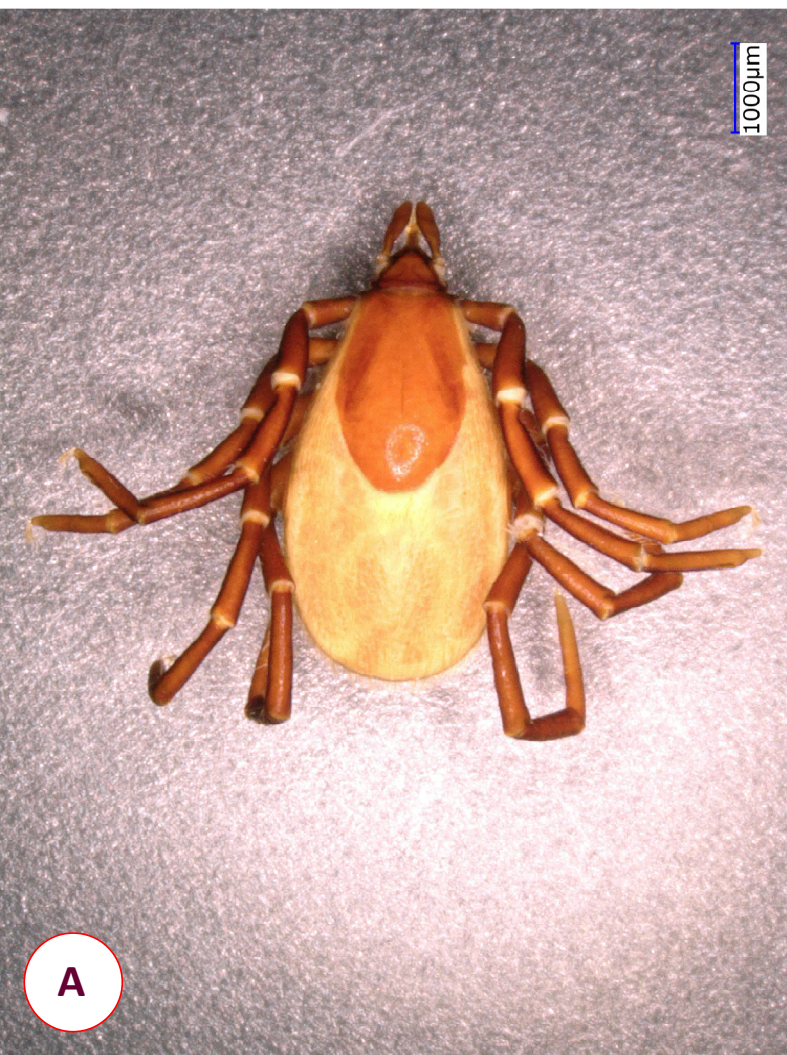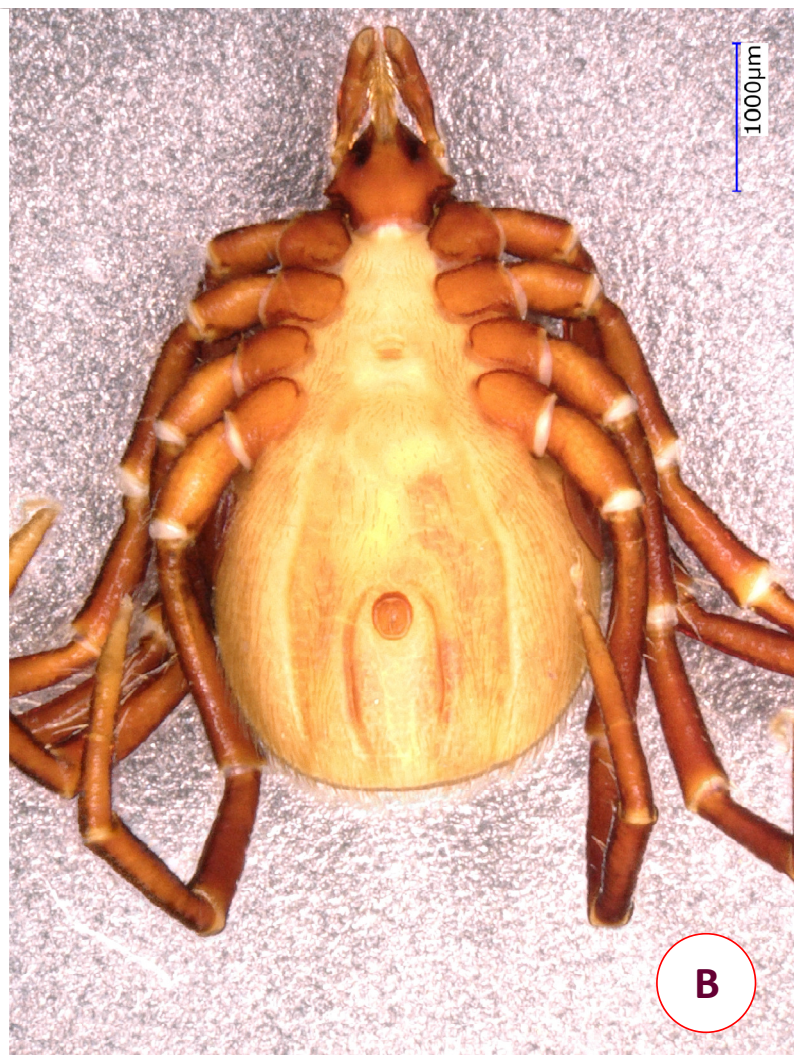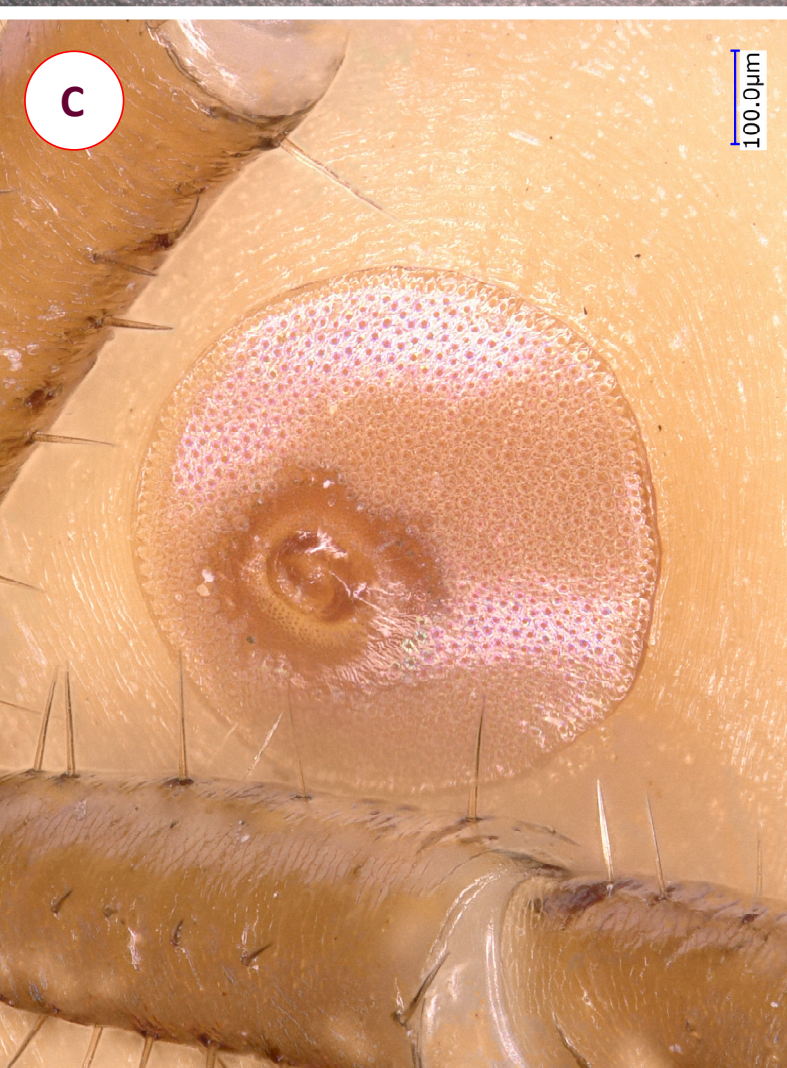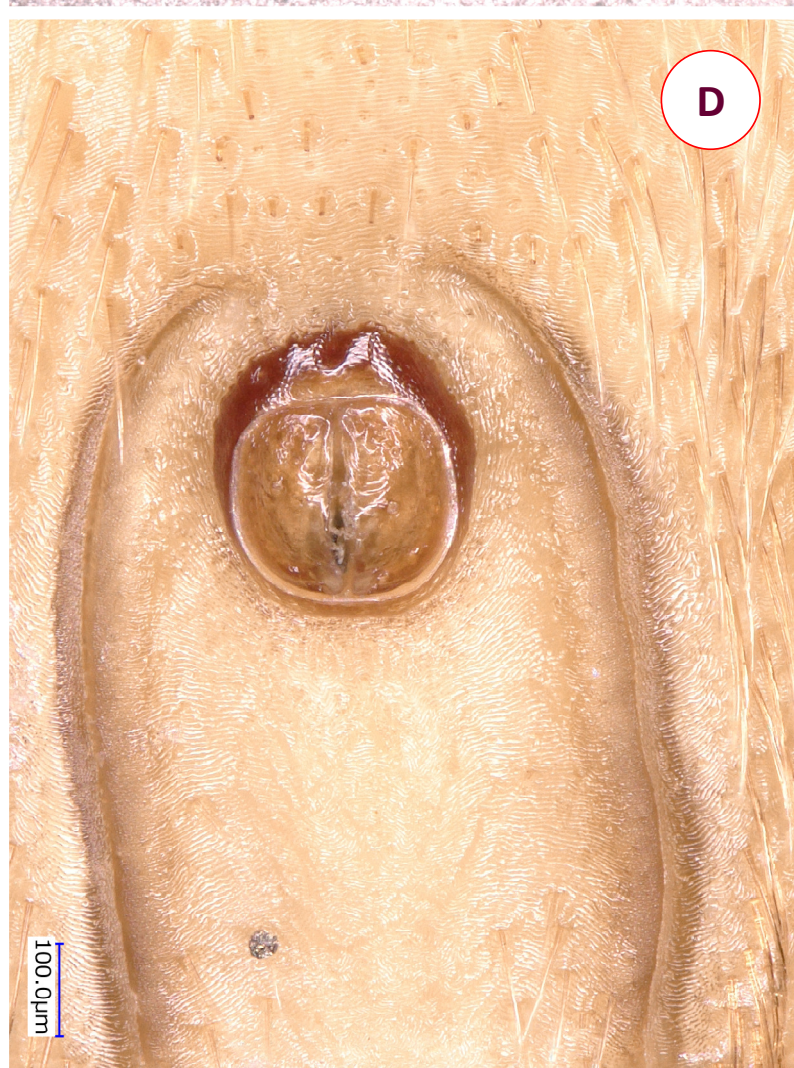

Supplement: Supplementary material 3 — Additional morphological details of Ixodescollaris [file zookeys-1180-001_article-108418__-s003.pdf]

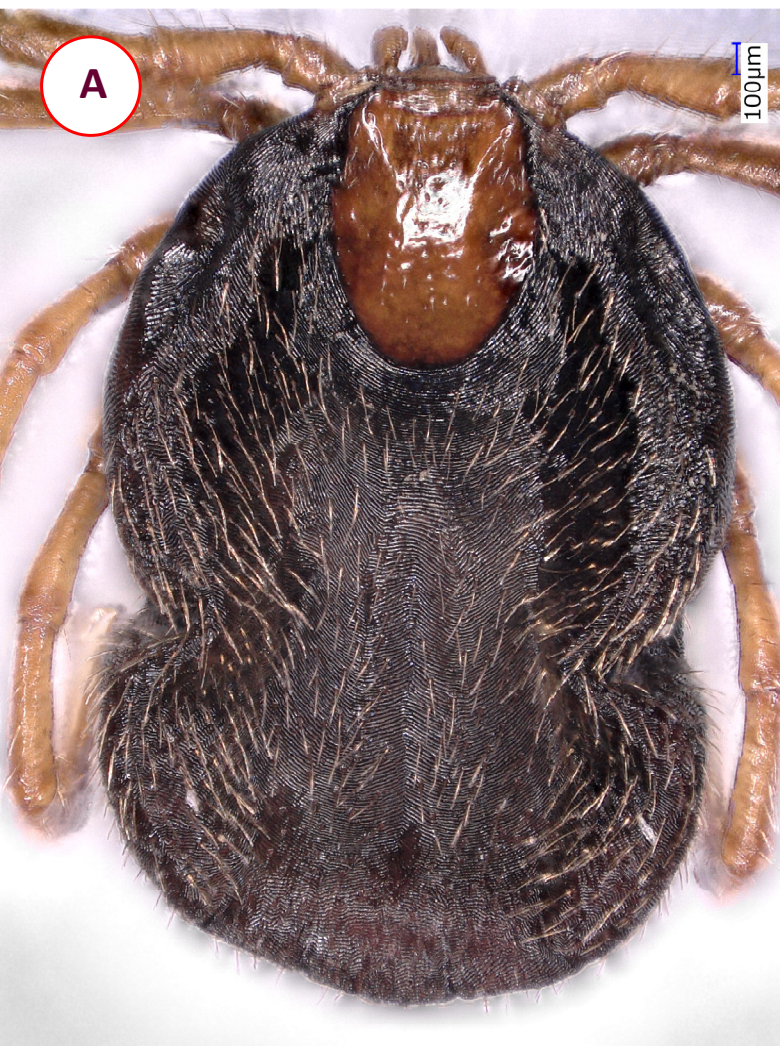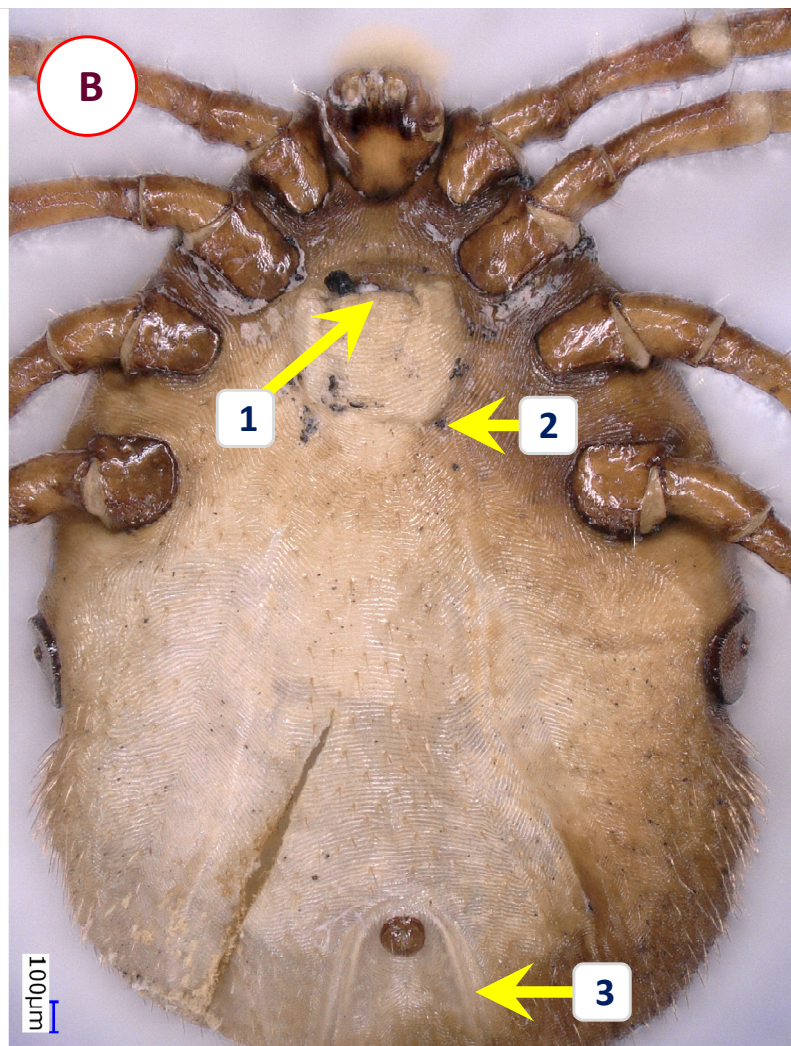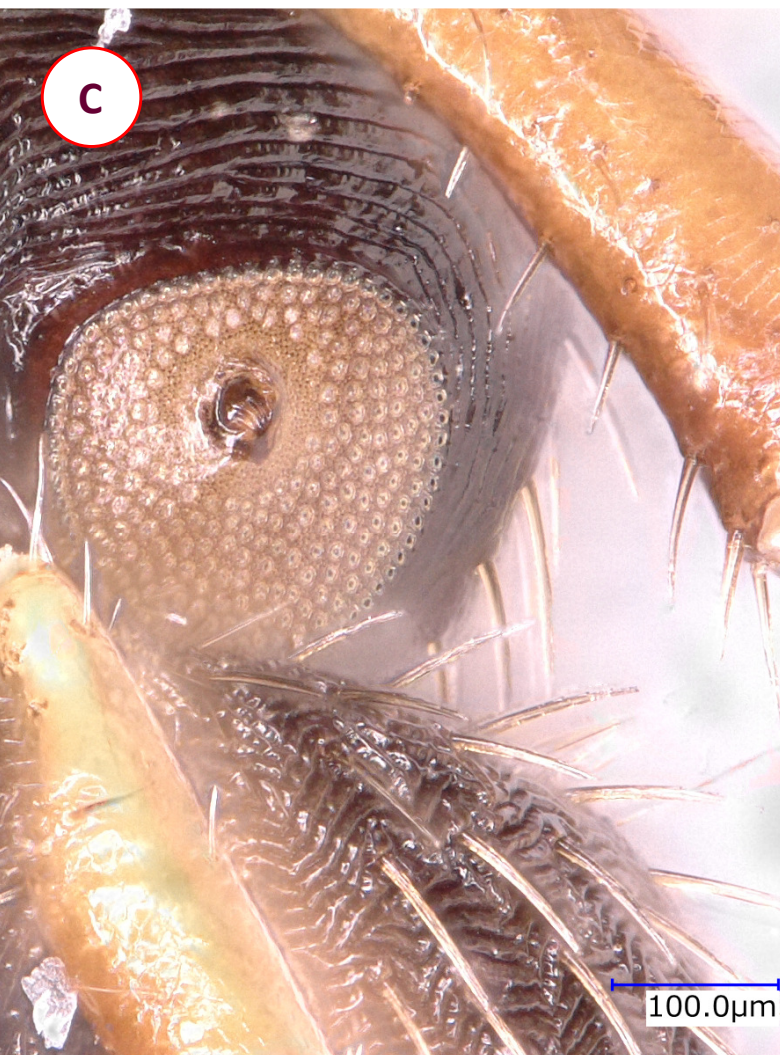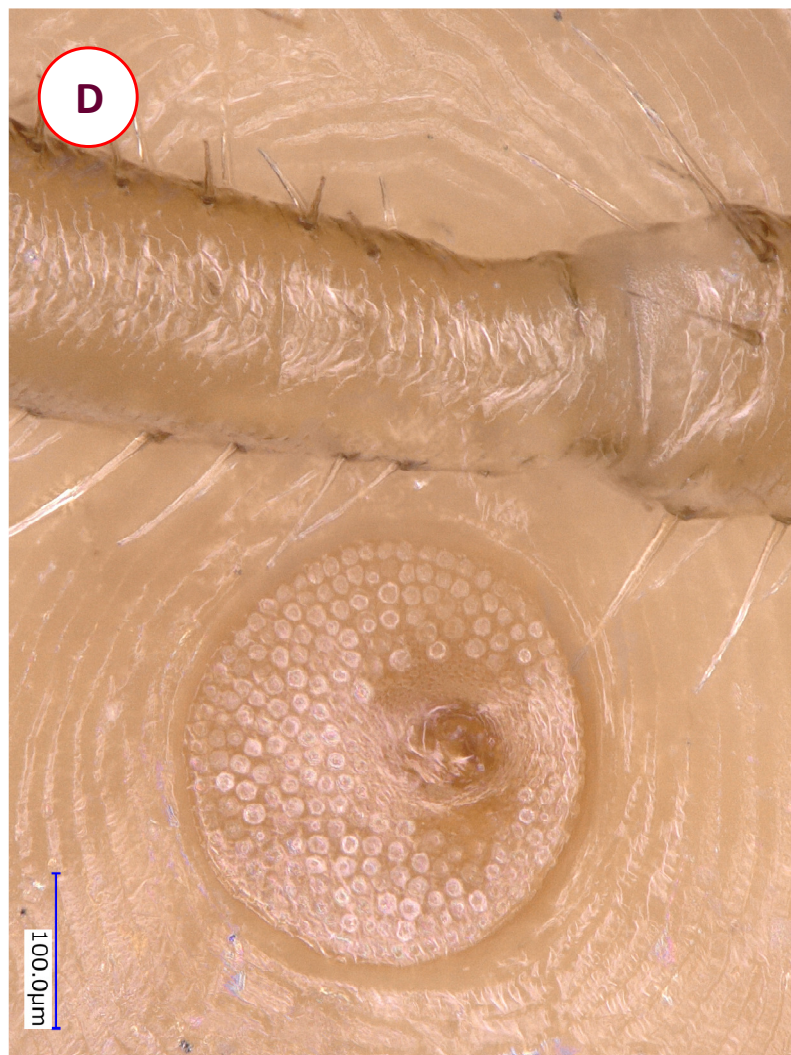

Supplement: Supplementary material 4 — Additional morphological details of Ixodesfuliginosus Hornok & Takano, sp. nov. [file zookeys-1180-001_article-108418__-s004.pdf]

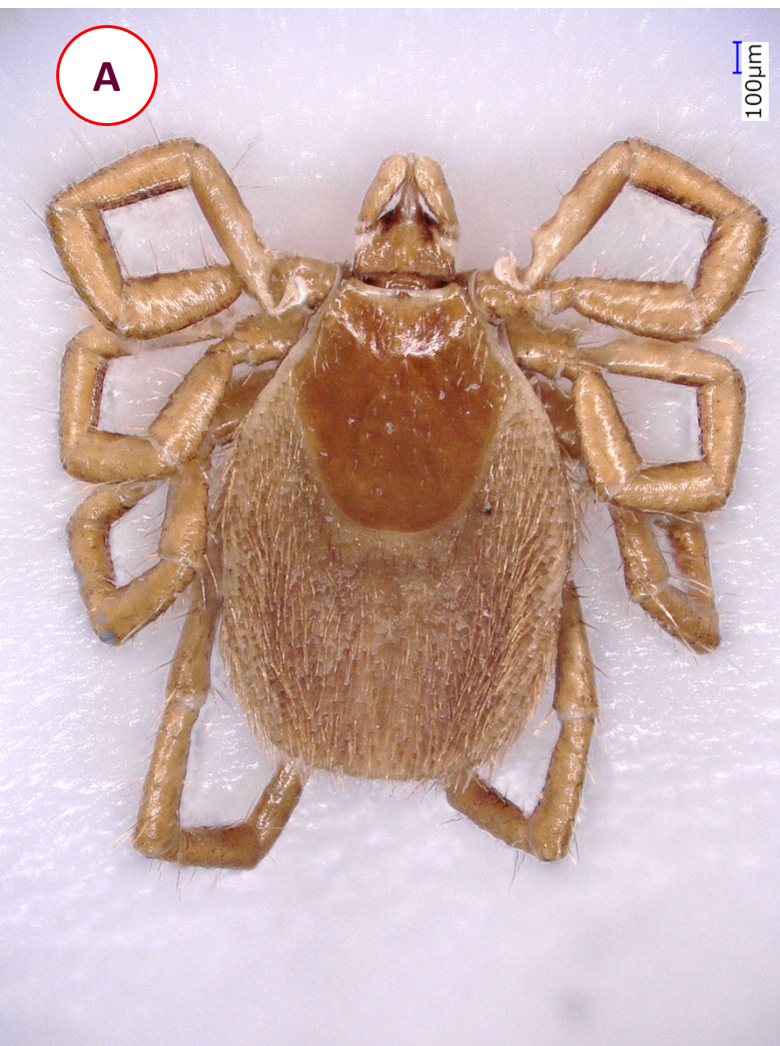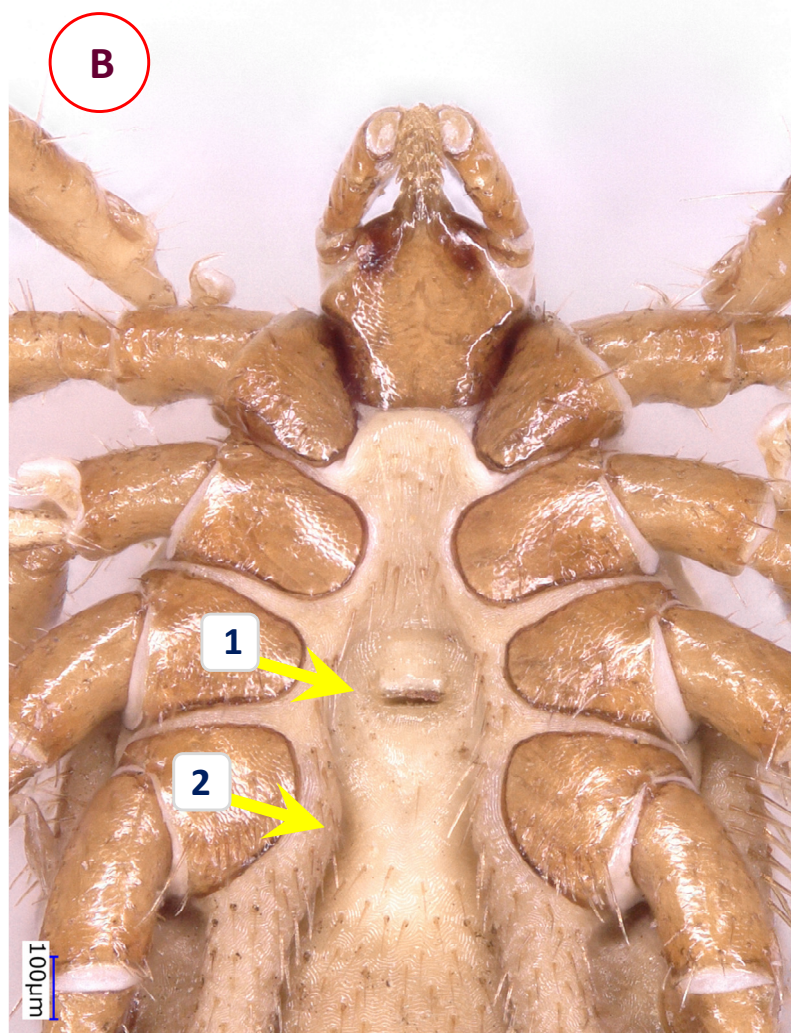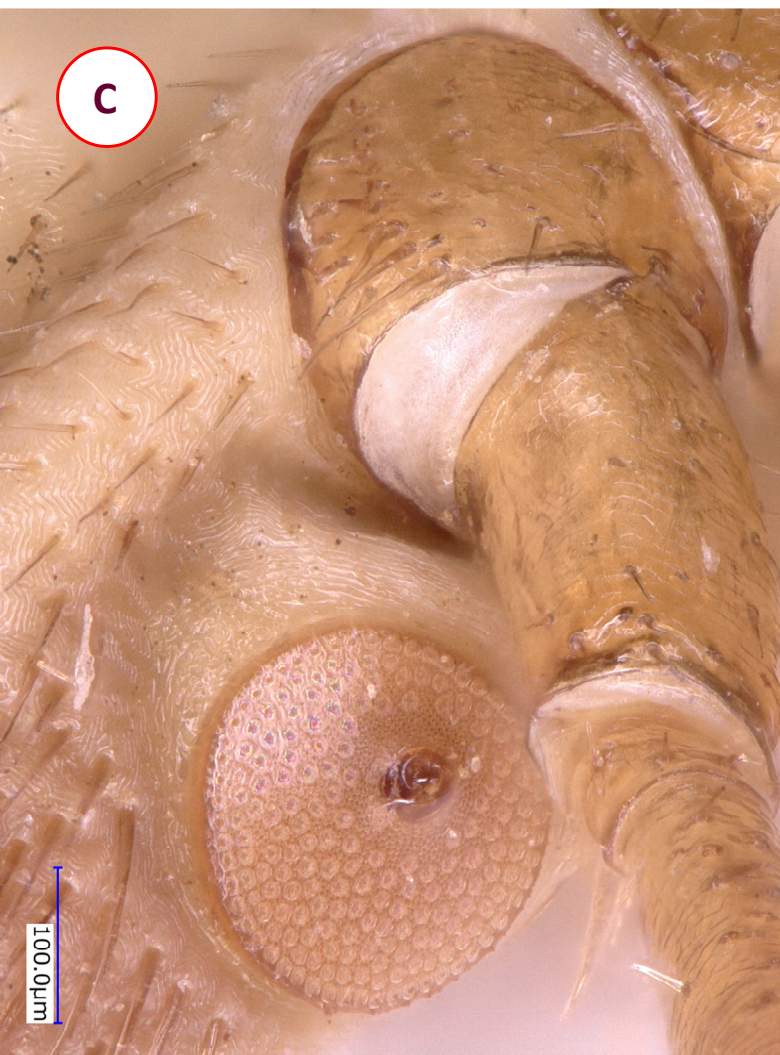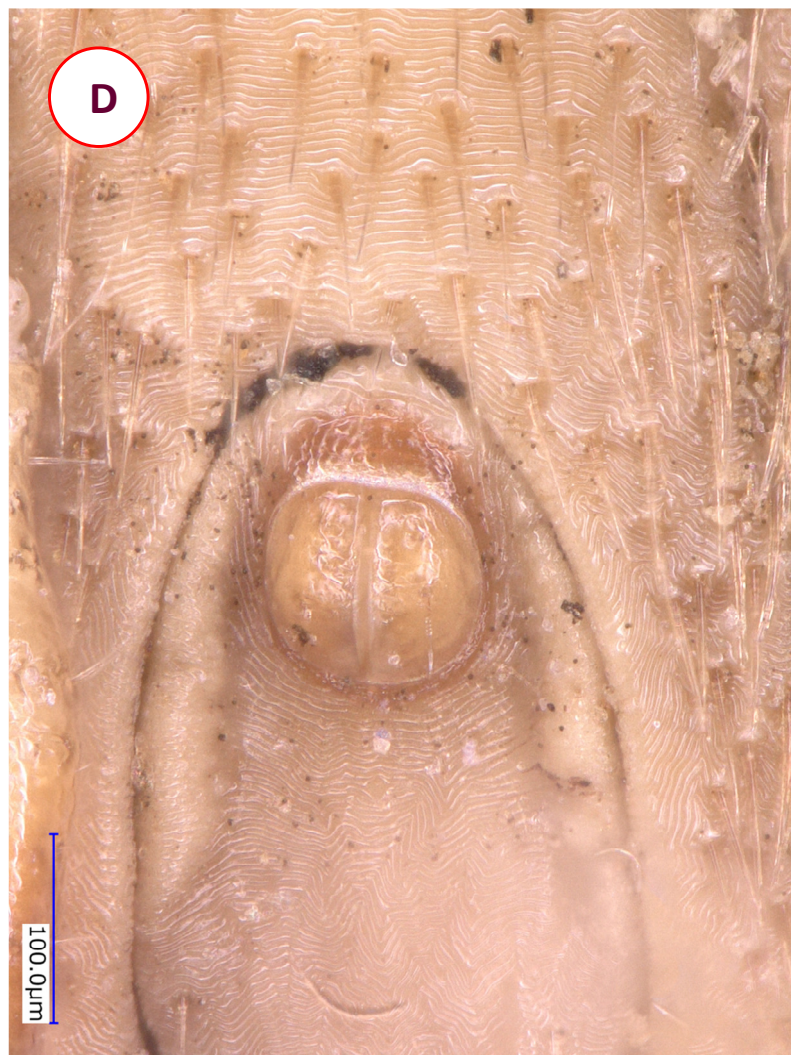

Supplement: Supplementary material 5 — Additional morphological details of Ixodessimplex [file zookeys-1180-001_article-108418__-s005.pdf]
